# Analytical ultracentrifugation with fluorescence detection system reveals differences in complex formation between recombinant human TNF and different biological TNF antagonists in various environments

**DOI:** 10.1080/19420862.2017.1297909

**Published:** 2017-03-03

**Authors:** Elena Krayukhina, Masanori Noda, Kentaro Ishii, Takahiro Maruno, Hirotsugu Wakabayashi, Minoru Tada, Takuo Suzuki, Akiko Ishii-Watabe, Masahiko Kato, Susumu Uchiyama

**Affiliations:** a Graduate School of Engineering, Osaka University, Yamadaoka, Suita, Osaka, Japan; b U-Medico Inc., Yamadaoka, Suita, Osaka, Japan; c Okazaki Institute for Integrative Bioscience, National Institutes of Natural Sciences, Higashiyama, Myodaiji, Okazaki, Aichi, Japan; d Division of Biological Chemistry and Biologicals, National Institute of Health Sciences, Kamiyoga, Setagaya-ku, Tokyo, Japan; e Sysmex Corporation, Murotani, Nishi-ku, Kobe-shi, Hyogo, Japan

**Keywords:** Adalimumab, analytical ultracentrifugation with fluorescence detection, etanercept, FcγR cell-reporter assay, immune complex, immunogenicity, infliximab, native mass spectrometry, size distribution, TNF

## Abstract

A number of studies have attempted to elucidate the binding mechanism between tumor necrosis factor (TNF) and clinically relevant antagonists. None of these studies, however, have been conducted as close as possible to physiologic conditions, and so the relationship between the size distribution of TNF-antagonist complexes and the antagonists' biological activity or adverse effects remains elusive. Here, we characterized the binding stoichiometry and sizes of soluble TNF-antagonist complexes for adalimumab, infliximab, and etanercept that were formed in human serum and in phosphate-buffered saline (PBS). Fluorescence-detected sedimentation velocity analytical ultracentrifugation analyses revealed that adalimumab and infliximab formed a range of complexes with TNF, with the major complexes consisting of 3 molcules of the respective antagonist and one or 2 molcules of TNF. Considerably greater amounts of high-molecular-weight complexes were detected for infliximab in human serum. The emergence of peaks with higher sedimentation coefficients than the adalimumab monomer as a function of added human serum albumin (HSA) concentration in PBS suggested weak reversible interactions between HSA and immunoglobulins. Etanerept exclusively formed 1:1 complexes with TNF in PBS, and a small amount of complexes with higher stoichiometry was detected in human serum. Consistent with these biophysical characterizations, a reporter assay showed that adalimumab and infliximab, but not etanercept, exerted FcγRIIa- and FcγRIIIa-mediated cell signaling in the presence of TNF and that infliximab exhibited higher potency than adalimumab. This study shows that assessing distribution profiles in serum will contribute to a more comprehensive understanding of the *in vivo* behavior of therapeutic proteins.

## Introduction

Since tumor necrosis factor (TNF) was established as one of the key mediators in the pathogenesis of several immune-mediated inflammatory diseases, the therapeutic effects of various TNF antagonists, including adalimumab (Ada), infliximab (Inf), and etanercept (Eta), have been demonstrated. Adalimumab is a human IgG1 monoclonal antibody (mAb),^1,2^ while infliximab is a chimeric mAb consisting of the TNF-binding site derived from the murine mAb A2 linked to the constant region of human IgG1.^3,4^ Etanercept is a recombinant fusion protein composed of a human IgG1 Fc fragment fused to the extracellular ligand-binding portion of the human TNF receptor (TNFR) p75.^5^

Adalimumab, infliximab, and etanercept are effective in treating rheumatoid arthritis, psoriatic arthritis, ankylosing spondylitis, and psoriasis.^2,4-8^ In contrast to etanercept, adalimumab and infliximab have demonstrated therapeutic effects in treating Crohn's disease and the clinical efficacy of the 2 has been estimated to be similar.^9-11^

Common side effects associated with TNF antagonists are usually mild and well tolerated, and the incidence and severity varies slightly between adalimumab, infliximab, and etanercept.^12^ Immunogenicity is another concern for TNF antagonists, and this can result in diminished efficacy and increased side effects. As with other pharmaceutical formulations, the therapeutic agents studied here can interfere with the immune system and might trigger immune responses leading to the formation of anti-drug antibodies (ADA). The incidence of immunogenicity in patients treated with TNF antagonist varies greatly among different studies. In psoriasis patients receiving infliximab or adalimumab, ADA have been observed at rates of 5.4–43.6% and 8.8–44.8%,^13^ whereas for the full range of Ada and Inf-treatable diseases the occurrence was 7–68%^14,15^ and 0.04–87%,^16^ respectively. In the case of etanercept, low levels of ADA were consistently measured and it was confirmed that they have no effect on the drug's efficacy and safety, whereas antibodies against infliximab and adalimumab were associated with decreased clinical response.^13^

Several hypotheses have been posited in an attempt to explain reported differences in the effectiveness of the 3 TNF antagonists in the treatment of various diseases. In addition to possible differences in pharmacokinetics and tissue distribution, the most likely explanation is related to differences in their ability to form complexes with TNF.

Each agent has been shown to have a strong intrinsic binding affinity for soluble TNF. The affinities, as determined by a surface plasmon resonance (SPR), were 30.4 pM for adalimumab, 27.3 pM for infliximab, and 11.8 pM for etanercept.^17^ In another study, a picomolar binding affinity of adalimumab to recombinant human TNF was confirmed using bio-layer interferometry (BLI) with an estimated dissociation equilibrium constant (K_d_) value of ∼4.6 pM.^18^ Recently, Ogura et al. reported the Kds for etanercept, infliximab, and adalimumab as 5.66 pM, 88.6 pM, and 277 pM, respectively, as measured by SPR.^19^

Size distributions and stoichiometry of complexes formed between TNF and 3 different antagonists have been characterized *in vitro*. A number of complexes with molecular weights (MWs) in the 600–5,800 kDa range formed between adalimumab and TNF. However, these larger complexes were found to be transient, and upon overnight incubation, a single stable complex with an MW of ∼598 kDa, consisting of 3 adalimumab and 3 TNF molecules, was identified.^20^ Similarly, in another study it was shown that adalimumab formed a variety of complexes with TNF with MWs of up to 4,000 kDa.^21^ In the case of infliximab, the MWs of the complexes were as high as 14,000 kDa.^21^ It has been suggested that the most thermodynamically stable complex is composed of 6 molcules of infliximab and 3 molcules of TNF.^22^ When infliximab was present in molar excess over TNF, complex formation between 3 molcules of infliximab and one TNF trimer was detected.^23^ In contrast to the mAbs, etanercept did not form large complexes; only 1:1 complexes with an apparent MW of 180 kDa were identified in the presence of excess TNF.^21-23^ When etanercept was present in excess, complex formation between 2 etanercept molecules and a single trimeric TNF (forming a 300 kDa complex) was observed.^21^ In another study, however, etanercept bound to TNF in a 1:1 stoichiometry, even when present in molar excess.^23^

The above-mentioned studies have clearly revealed that different TNF antagonists form various types of complexes. These findings, however, cannot be easily extrapolated to the *in vivo* environment to explain differences in the clinical efficacy of different TNF antagonists. Size-exclusion chromatography (SEC) coupled with light scattering (LS) or refractive index (RI) detectors and dynamic light scattering (DLS) techniques that were used in these studies require relatively simple solutions where only the molecule of interest and its interaction partner are present. Additionally, analysis is often restricted by the small number of amenable solvents, which are usually limited to general solvents such as phosphate buffers. Nevertheless, Demeule et al. showed that different complexes between a recombinant humanized mAb and its antigen can form in serum and phosphate-buffered saline (PBS).^24^ Due to technical limitations, characterization of TNF-antagonists complexes was only performed in the micromolar concertation range.

The present study aimed to reveal binding characteristics of adalimumab, infliximab, and etanercept to recombinant human TNF under near-physiologic concentrations and solution environment conditions. The sedimentation velocity analytical ultracentrifugation (SV AUC) with absorbance (UV) detection conducted at the micromolar range showed that infliximab formed the largest complexes, followed by adalimumab, and the smallest complexes were detected with etanercept, which is consistent with previously reported findings. The next target drug concentration (25 nM) was chosen based on actual serum concentrations measured in patients.^2,4,5^ Complexes that formed in the presence of TNF at 3 concentrations from 2.5 to 25 nM (assuming TNF is in its trimer form) were analyzed using a fluorescence detection system (FDS) coupled with SV AUC. AUC has become a widely accepted method for accurate determination of size distributions of macromolecules in solution.^25-28^ Compared with previously used SEC and DLS methods, AUC is capable of providing higher resolution, is applicable for a virtually unlimited variety of solvent compositions, and quantification is not affected by the presence of large aggregates.^29-32^ When coupled with the recently developed FDS, AUC has the additional advantage of allowing measurements to be performed in nanomolar and picomolar concentration ranges.^33-36^ SV measurements using current commercially available FDS require chemical labeling of the target macromolecule with fluorescent labels with excitation maxima at 488 nm and emission at 505–565 nm. From several suitable fluorescent dyes, we chose Alexa Fluor 488 owing to its high labeling efficiency.^37^ To confirm the integrity and TNF-binding capacity of Alexa Fluor 488-labeled antagonists, SV experiments were first performed in PBS where ideal sedimentation behavior is usually observed. Additionally, using the unprecedented ability of FDS to detect sedimentation in highly non-ideal, crowded solution environments, SV experiments were conducted in human serum.^34,38^ To assign the peaks yielded by conventional continuous *c(s)* distribution modeling with SEDFIT,^39^ SV data were further analyzed using the hybrid local continuous distribution and global discrete species model of SEDPHAT.^40^ The stoichiometries of the derived complexes were corroborated by native mass spectrometry (MS) measurements. A dependence of sedimentation coefficient distribution on the TNF mixing ratio was observed. To explain this, a theory was proposed whose trends were confirmed by simulation data generated using adalimumab-Fab-TNF dissociation constant of 11.6 nM as estimated by isothermal titration calorimetry (ITC). Based on the differences in complex formation revealed by AUC and the different abilities to activate FcγRIIa and FcγRIIIa demonstrated using a reporter cell assay, a possible mechanism responsible for the differences in biological activity of various TNF antagonists is discussed.

## Results

### Interaction analysis in PBS

Recombinant human TNF purified from yeast or *E. coli* was shown to be present in its trimeric form in previous crystallographic^41^ and AUC studies.^42^ To confirm the oligomeric state of human TNF produced recombinantly in the baculovirus expression system used in this study, UV-SV AUC was performed using samples at a concentration of 2 μM. Sedimentation coefficient distributions revealed a main peak, amounting to more than 96% of the total signal intensity, with a sedimentation coefficient *s_20,w_* of 3.9 S and an estimated MW of 52 kDa, consistent with the calculated molecular weight of a trimer (Fig. S1A). Similar results were obtained in the control experiments performed with recombinant human TNF prepared using an *E. coli* expression system (Fig. S1B). Furthermore, FDS-SV with fluorescently labeled baculovirus-expressed TNF confirmed that it exists primarily as a stable, soluble trimer over a 2.5–25 nM concentration range in both PBS (Fig. S1C) and human serum (Fig. S1D). Accordingly, we used this concentration for trimeric TNF throughout the remainder of the manuscript.

**Figure 1.**
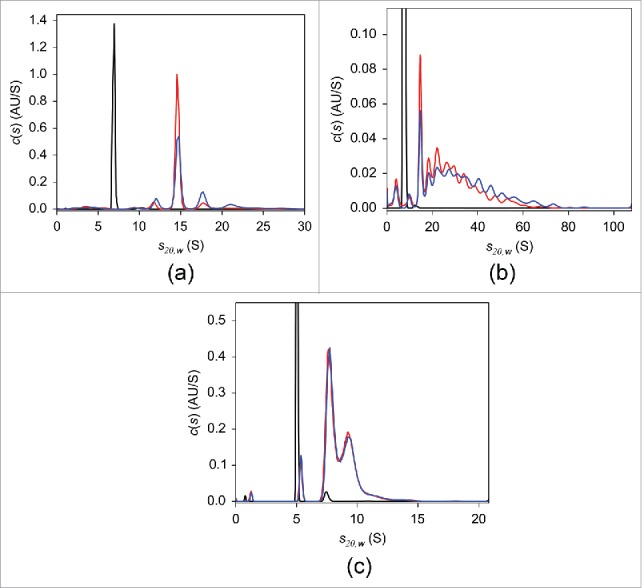
UV-SV with 2 μM adalimumab (a), infliximab (b), and etanercept (c). The c*(s)* distributions in the absence of TNF (black) and of the 1:1 molar mixtures of the respective antagonist:TNF incubated at 20°C for 2 hours (blue) or at 37°C overnight (red) are shown.

As mentioned above, a preparatory step to fluorescently label the TNF antagonists is required for the FDS-SV experiments. Prior to conjugation of fluorescent probe, the ability of native, non-labeled adalimumab, infliximab, and etanercept to form complexes with TNF was evaluated using UV-SV (Fig. 1). At a concertation of 2 μM, in the absence of TNF, all antagonists revealed single-peaked *c(s)* distributions. The peak sedimentation coefficient *s_20,w_* and estimated MW were 6.9 S and 144 kDa for adalimumab and 7.4 S and 141 kDa for infliximab, consistent with the monomeric form of the respective antibody. In the case of etanercept, a major peak was at 5.0 S with an estimated MW of 119 kDa, which is less than the 150 kDa expected for monomeric etanercept.^5^ The frictional ratio value derived from the analysis was 2.0, in contrast to 1.4 for adalimumab and infliximab, indicating that etanercept has a more asymmetric shape. It is thought that the main underlying reason for the expanded conformation of etanercept is heavy glycosylation in the Fc region.^43^ We concluded that the elongated structure of etanercept affected its sedimentation behavior and led to a decreased apparent sedimentation coefficient, which in turn resulted in an underestimated MW. Neither experimental nor structural data allowing for hydrodynamic calculation of sedimentation coefficient are available, which precludes unambiguous identification of the 5.0 S peak. We tentatively concluded that this peak corresponds to the monomer. When TNF at equimolar concentrations was added to antagonist solutions, peaks with sedimentation coefficients larger than those of the monomeric antagonists corresponding to complexes were observed. The weight-average sedimentation coefficients indicated that the largest complexes with TNF were formed by infliximab, whereas considerably smaller complexes were detected in adalimumab and etanercept mixtures (Table S1). Similar results were obtained using DLS, where the average hydrodynamic radius of each antagonist in solution increased with increasing molar ratios of added TNF, indicating formation of antagonist-TNF complexes. The most prominent increase was detected for infliximab, followed by adalimumab and etanercept (Fig. S2). In agreement with the previously published SEC findings,^20^ our UV-SV results indicated that overnight incubation of adalimumab-TNF mixture at 37°C led to the formation of a single major complex with *s_20,w_* of 14.6 S (Fig. 1A). Even though a slight decrease in populations of complexes with large sedimentation coefficients was observed following overnight incubation, in contrast to adalimumab, infliximab-TNF complexes exhibited broad continuous sedimentation coefficient distributions (Fig. 1B). No substantial changes in the *c(s)* distribution of etanercept-TNF complexes were detected in response to overnight incubation (Fig. 1C).

Next, we labeled adalimumab, infliximab, and etanercept with Alexa Fluor 488 fluorescent dye. Previous studies have clearly demonstrated that the FDS is capable of providing accurate data that are consistent with measurements conducted at micromolar concentrations using conventional absorption optics.^44^ Therefore, the characterization of labeled TNF antagonists was directly performed at nanomolar concentrations using FDS.

To evaluate whether the covalently attached fluorescent probe affected protein structure or stability, and to confirm that the fluorescently labeled antibodies retained TNF-binding capacity, adalimumab, infliximab, and etanercept at 25 nM were subjected to FDS-SV experiments in PBS. To prevent non-specific adsorption of protein to the surfaces, such as centerpiece walls and windows, 0.1 mg/mL lysozyme was added to the solutions.

**Figure 2.**
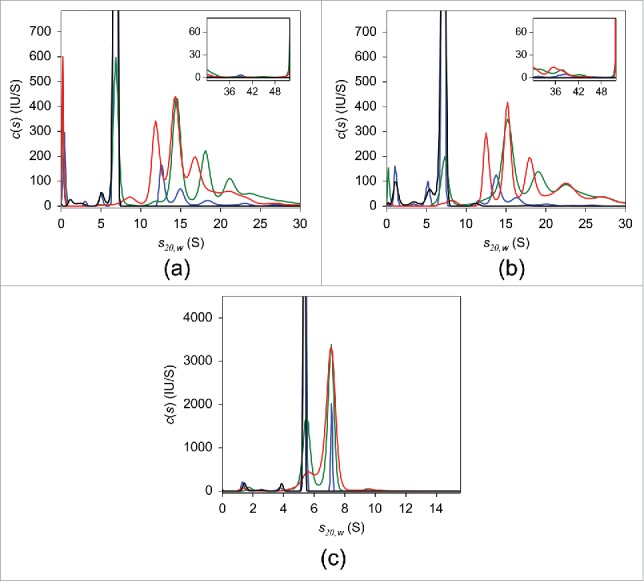
FDS-SV with 25 nM adalimumab (a), infliximab (b), and etanercept (c) in the presence of varying concentrations of TNF in PBS. The c*(s)* distributions in the absence of TNF (black) and of the 10:1 (blue), 2:1 (green), and 1:1 (red) molar mixtures of the respective antagonist:TNF are shown.

The results of SV analysis of adalimumab are shown in Fig. 2A. Similar to the UV-SV findings, in the absence of TNF, a single peak with sedimentation coefficient *s_20,w_* of 6.8 S and estimated MW of ∼150 kDa, consistent with monomeric adalimumab, was observed in the *c(s)* distribution. Mixing adalimumab and TNF at a molar ratio of 10:1 induced an ∼10% reduction in uncomplexed adalimumab. When the mixing ratio was 2:1 adalimumab to TNF, the level of uncomplexed adalimumab was less than 50%. In the presence of an equimolar concentration of TNF, no free monomeric adalimumab was observed and the major adalimumab:TNF complexes were detected at 8.2 S, 11.9 S, 14.4 S, and 17.4 S (Table 1). In equimolar mixtures of infliximab and TNF, the major complex peak was at 15.2 S (Fig. 2B). A nearly exclusive 7.0 S peak appeared in the case of etanercept (Fig. 2C). The consistency between these and UV-SV results indicates that there was no considerable effect of fluorescent labeling on the interaction of antagonists with TNF. Detailed FDS-SV results obtained for infliximab and etanercept are described further below.

**Table 1.** TNF-antagonist complexes detected using native MS and AUC FDS-SV.

	Native MS					
	MW (Da)			AUC FDS-SV		
TNF antagonist	measured	calculated	Stoichiometry	*s_20,w_* (S)	MW_app_ (kDa)	Stoichiometry
adalimumab	203,667	203,707	1:1	8.2	222	1:1
	352,252	351,854	2:1	11.9	383	2:1
	408,047	407,415	2:2	14.4	508	3:1
	556,656	555,561	3:2	17.4	652	3:2
infliximab	204,402	204,408	1:1	8.0	190	1:1
	353,474	353,297	2:1	12.6	377	2:1
	409,362	408,816	2:2	15.2	502	3:1
	559,262	557,664	3:2	18.2	654	3:2
etanercept	190,306	189,307	1:1	7.0	163	1:1

To compare the sizes of various complexes detected in the *c(s)* distributions of mixtures of the different antagonists and TNF, the weight-average sedimentation coefficients were calculated for each experimental condition (Table 2). The resulting values indicated that infliximab was capable of forming the largest soluble complexes with TNF, whereas the smallest complexes were detected in etanercept-TNF mixtures, in agreement with DLS results.

**Table 2.** The weight-average sedimentation coefficients calculated for each experimental condition.

		*s_20,w_* (S)	
TNF antagonist	TNF concertation (nM)	PBS	human serum^1^
adalimumab	0	6.8	7.6
	2.5	7.8	9.2
	12.5	15.7	23.8
	25	15.5	20.2
infliximab	0	7.1	9.4
	2.5	8.9	12.7
	12.5	18.4	36.0
	25	19.0	47.0
etanercept	0	5.2	4.6
	2.5	5.4	4.8
	12.5	6.4	5.8
	25	6.8	6.4

1 The signal originating from the HSA-bilirubin complex as measured in SV experiments performed with human serum alone was subtracted from the total fluorescent signal obtained by integrating the area under the *c(s)* distribution of antagonist-TNF mixtures.

To evaluate the stoichiometric ratio of adalimumab to TNF in the 4 major complexes, SV data were further analyzed in SEDPHAT using the hybrid local continuous distribution and global discrete species model. To define parameter values for discrete species, each of the 4 above-mentioned sedimentation coefficients was combined with calculated MWs, using 148 kDa for adalimumab and 55 kDa for TNF, according to values specified in the product monograph^2^ and measured by MS, respectively. The continuous distribution segment range was set to 20–50 S. Several models comprising different reasonable combinations of sedimentation coefficients and MWs reflecting different stoichiometries of adalimumab-TNF complexes were tested (Table S2). Assuming that the minimum root mean-square deviation (rmsd) indicates the model that best fits the data, the major complexes are likely to correspond to (Ada)_1_(TNF)_1_, (Ada)_2_(TNF)_1_, (Ada)_3_(TNF)_1_, and (Ada)_3_(TNF)_2_. Three other acceptable models, for which the rmsd values do not exceed the critical value, are shown in Table S2.

**Figure 3.**
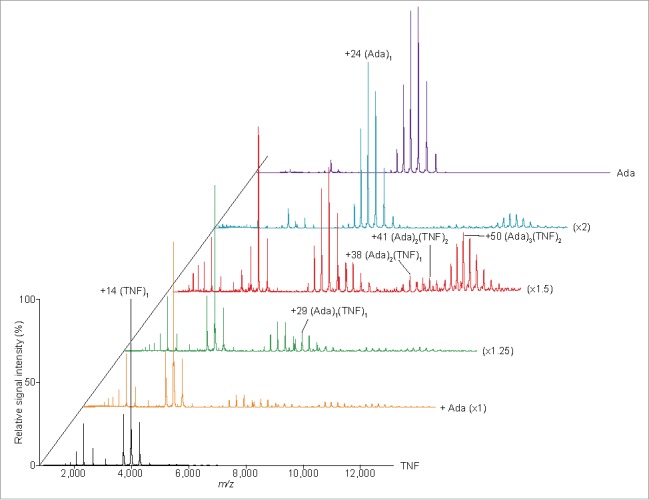
Mass spectra of the TNF-adalimumab complexes. Mass spectra of the mixtures containing trimeric TNF:adalimumab at molar ratios of 1:0 (black), 1:1 (orange), 1:1.25 (green), 1:1.5 (red), 1:2 (cyan), and 0:1.5 (purple) are shown.

Peak assignments based on MWs derived from SV data remain ambiguous because a relatively low degree of accuracy is anticipated for continuous distributions, although for single-peak distributions the estimations can be expected to fall within 10% of the true MW value.^40,45^ Conversely, MS under non-denaturing conditions has been shown to be a powerful approach for estimating the stoichiometry of non-covalently bound protein complexes.^46-49^ Therefore, to verify our AUC results, the stoichiometry of adalimumab-TNF complexes was evaluated using MS under non-denaturing conditions (Fig. 3). (Ada)_1_(TNF)_1_, (Ada)_2_(TNF)_1_, (Ada)_2_(TNF)_2_, and (Ada)_3_(TNF)_2_ complexes were detected in experiments with 2–4 µM adalimumab and 2 µM TNF, corroborating the peak assignment determined by SV analysis (Table 1).

In the 25 nM equimolar mixture of adalimumab and TNF, the area under the 11.9 S-peak was larger compared with the 2:1 adalimumab:TNF mixture. In addition, an 8.2 S-peak only appeared in the equimolar mixture and was not detected when adalimumab and TNF were mixed at a 2:1 molar ratio. Similar results were obtained in experiments performed with 50 nM (Fig. S3A) and 100 nM of adalimumab (Fig. S3B). When adalimumab and TNF were mixed at molar ratios of 2:1, 1:1, and 1:2, major peaks were detected at 11.9 S and 14.4 S. Again, a smaller peak at 8.4 S was detected only in the presence of equimolar or excess amounts of TNF. In the *c(s)* distributions of the 2:1 molar mixtures of adalimumab:TNF, the largest major complex was detected at 18.2 S, whereas with ratios of 1:1 and 1:2 molar this peak was absent, and the 17.0 S-peak was observed instead. As discussed above, the 17.0 S peak is likely to represent (Ada)_3_(TNF)_2_, whereas the 18.2 S peak corresponds to a larger complex. These results suggest that a molar excess of TNF over adalimumab results in the formation of smaller complexes.

**Figure 4.**
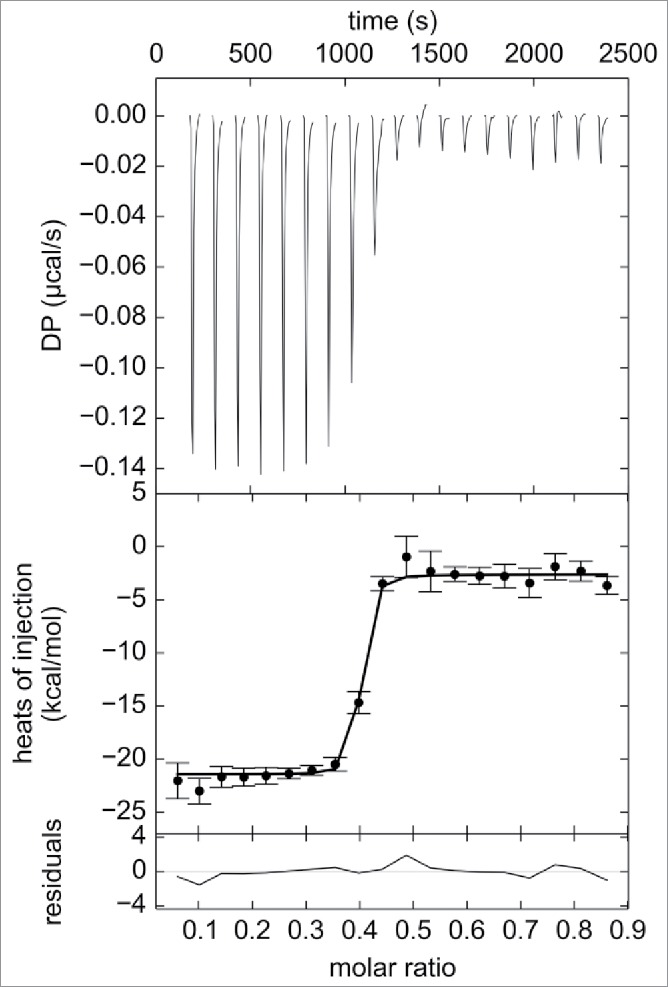
ITC analysis of direct titration of 39.8 μM trimeric TNF into 9.8 μM adalimumab-Fab. Raw heat changes (top panel), normalized heat changes with best-fit values (solid line) (middle panel), and residuals of the fit (bottom panel) are shown. Non-linear regression analysis of the curves using A + B + B + B <-> AB + B + B <-> ABB + B <-> ABBB binding model yielded the K_d_ of 11.6 ± 1.7 nM and ΔH values of 11.6 ± 1.7 nM and -6.4 ± 0.1 kcal/mol, respectively, as determined from triplicate measurements. Representative profile from triplicate measurements is shown.

To verify that the observed changes in complex sizes were due to changes in the relative concentrations of adalimumab and TNF, numerical simulations using the Solver add-in for Microsoft Excel were performed.^50^ It was assumed that one adalimumab (antibody, Ab) molecule is capable of binding up to 2 TNF (antigen, Ag) molecules, and one Ag molecule is capable of binding up to 3 Ab molecules in a sequential manner. The maximum number of Ab molecules participating in the interaction was limited to 3, which is supported by the MS results obtained in this study. The dissociation constants of the 9 possible AbAg complexes AbAg, AbAg_2_, Ab_2_Ag, Ab_2_Ag_2_, Ab_2_Ag_3_, Ab_3_Ag, Ab_3_Ag_2_, Ag_3_Ag_3_, and Ab_3_Ag_4_ were expressed as functions of molar fractions of each of the components in the solution. Together with the total Ab and Ag concentration constraints, the derived equations were used to determine the best-fit populations of each AbAg complex in the mixtures containing 25 nM Ab and 2.5–100 nM Ag by nonlinear regression analysis. All 9 dissociation constant values were assumed to be equal to that derived from the ITC analysis of direct titration of TNF into an adalimumab-Fab solution. The raw heat signals were integrated to generate a plot of Δ*H* kcal/mol of injected TNF versus the molar ratio of TNF/Fab, as displayed in the middle panel of Fig. 4. Non-linear regression analysis of the curves yielded the dissociation constant of 11.6 ± 1.7 nM, as determined from triplicate measurements. ITC experiments were also performed using full-length adalimumab, and a somewhat smaller value of 2.2 nM was obtained (Fig. S4).

**Figure 5.**
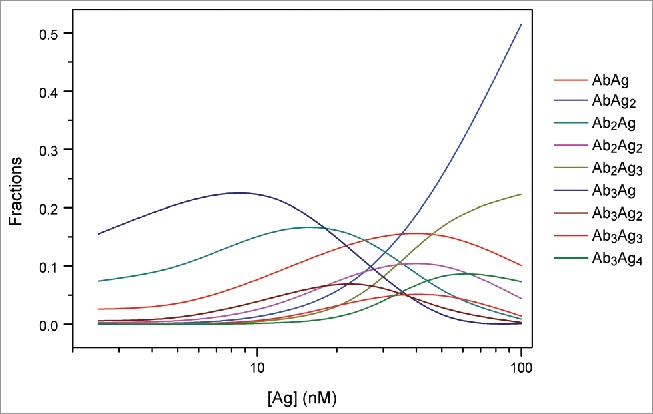
Simulation analysis of the populations of each AbAg complex in an Ab-Ag mixture. Simulations were performed for the mixtures containing 25 nM Ab and 2.5–100 nM Ag using K_d_ of 11.6 ± 1.7 nM derived from the ITC analysis of direct titration of TNF into an adalimumab-Fab solution.

With the exception of the Ab_3_Ag_4_ complex, the simulations predicted dependence similar to that observed in AUC, indicating that at equimolar and higher concentrations of TNF, populations of complexes containing 3 molcules of adalimumab decreased, accompanied by an increase in the amount of complexes composed of one or 2 adalimumab molecules (Fig. 5).

Next, we performed FDS-SV with 25 nM of infliximab (Fig. 2B). In the absence of TNF, a major peak with *s_20,w_* of 7.1 S and estimated molecular weight of 152 kDa was detected in the *c(s)* distribution, indicating that infliximab exists primarily in its monomeric form, in agreement with the UV-SV result. At a 10-fold molar excess of infliximab over TNF, a few peaks were detected, with the major peak at 13.9 S. In the mixtures containing infliximab:TNF at molar ratios of 2:1 and 1:1, the major peak was at 15.2 S with an apparent MW of ∼500 kDa, which suggests that this peak may correspond to (Inf)_3_(TNF)_1_ or (Inf)_3_(TNF)_2_. Similar to adalimumab, at equimolar infliximab:TNF, peaks with smaller sedimentation coefficients of 8.0 S and 12.6 S were observed. It should be noted that for each infliximab:TNF mixing ratio, the weight-average sedimentation coefficients were higher than those for adalimumab:TNF mixture (Table 2).

To assign the peaks to specific complexes, SV data for the equimolar mixture were analyzed using the hybrid local continuous distribution and global discrete species model of SEDPHAT. The 8.0 S, 12.6 S, 15.2 S, and 18.2 S peaks were designated discrete species, and the continuous distribution segment range was set at 20–100 S. Different models were generated by sequentially assigning a reasonable MW calculated for infliximab-TNF complexes with different stoichiometries to each discrete peak, using MWs of 149 kDa and 55 kDa for infliximab and TNF, respectively. The model comprising (Inf)_1_(TNF)_1_, (Inf)_2_(TNF)_1_, (Inf)_3_(TNF)_1_, and (Inf)_3_(TNF)_2_ complexes provided the best fit for the experimental databased on rmsd. Statistically indistinguishable fits with rmsd values less than the critical value were achieved using 5 other models, which are summarized in Table S3. Thus, the 8.0 S peak likely represents (Inf)_1_(TNF)_1_ and the 12.6 S peak corresponds to (Inf)_2_(TNF)_1_, which is highly consistent with the MS results (Fig. S5; Table 1).

Finally, we performed FDS-SV on 25 nM etanercept alone and in the presence of varying concentrations of TNF (Fig. 2C). In the absence of TNF, the sedimentation coefficient distribution had a major peak with *s_20,w_* of 5.3 S and an estimated molecular weight of 114 kDa, which was tentatively attributed to monomeric etanercept, similar to the UV-SV result. Increasing amounts of added TNF resulted in an increased 7.0 S peak area with an apparent MW of 163 kDa. Given an estimated MW of 114 kDa for the etanercept monomer and 55 kDa for the trimeric form of TNF, the 7.0 S peak probably corresponds to an (Eta)_1_(TNF)_1_ complex, consistent with previously published studies.^22,23^ This assignment is supported by the MS results obtained for etanercept, which indicated the presence of only 1:1 complexes irrespective of TNF concentration, in contrast to adalimumab and infliximab (Table 1).

### Interaction analysis in human serum

Human serum is a highly complex medium that contains a substantial amount of proteins, with human serum albumin (HSA) being the most abundant, followed by immunoglobulin (IgG). HSA is a highly soluble and stable protein that has a molecular weight of 66.5 kDa and is present in serum at a concentration of ∼40 mg/mL.^51^ In previous studies, FDS-SV AUC experiments performed with serum alone consistently showed a peak at ∼4 S, which was attributed to HSA bound to a natively fluorescent low-molecular-weight molecule, such as flavin or heme oxidation product.^24^ Recently, based on the fact that it has fluorescence excitation and emission spectra allowing detection with FDS, Lamola et al. concluded that this peak instead represents an oxidized bilirubin–HSA complex.^52^ Similar results were obtained in the present study, where SV experiments performed with human serum alone revealed the presence of a peak with *s_20,w_* of ∼4.0 S. For 25 nM antagonists suspended in human serum, at an optimized gain setting, approximately half of the total fluorescent signal was due to albumin. Similar contributions from albumin were reported for 10 nM anti-IgE IgG in human serum,^24^ whereas in another study, the HSA signal was insignificant.^53^ The intensity of the HSA-related signal can vary due to different amounts of bilirubin–HSA complex in serum from different sources or with storage times. In general, to minimize oxidation, and hence the amount of this unwanted contaminant, it is recommended that the freshest possible serum be used in FDS-SV studies.^53^

In the previous attempts to apply FDS-SV to proteins in serum, imperfections of the fit arising from non-ideality or other unknown origin were encountered.^34,38^ In our study, even though the fitted curves showed some deviations early in the run, overall they matched the shape of the experimental data with considerable accuracy, and the resulting rmsd did not exceed 1% of the total loading signal (Fig. S6). The *c(s)* analysis of SV data acquired for 25 nM of adalimumab, infliximab, and etanercept in the presence of various amounts of TNF in human serum yielded frictional ratio values between 2.5 and 3.5, which were higher than those observed in PBS. It has been reported that larger frictional ratio values cause peak broadening.^45,54^ Consistent with this finding, the respective derived *c(s)* distributions from analysis of data acquired in human serum exhibited broader peaks than in PBS.

**Figure 6.**
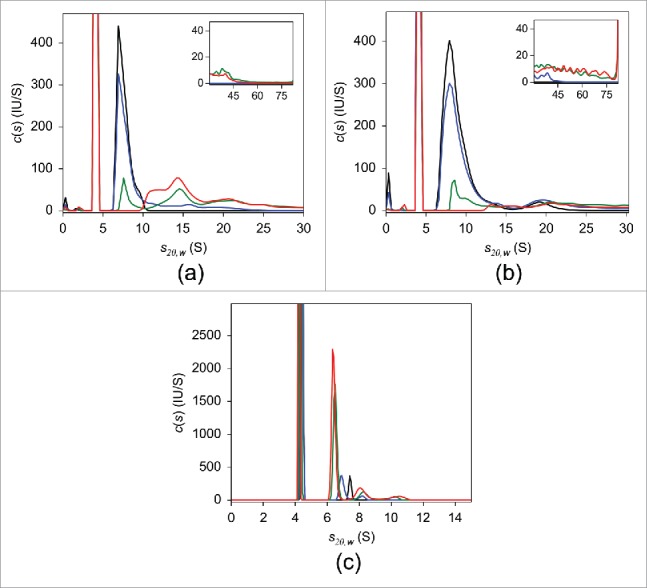
FDS-SV with 25 nM adalimumab (a), infliximab (b), and etanercept (c) in the presence of varying concentrations of TNF in human serum. The* c(s)* distributions in the absence of TNF (black) and of the 10:1 (blue), 2:1 (green), and 1:1 (red) molar mixtures of the respective antagonist:TNF are shown.

Under conditions where no TNF was exogenously added, a major peak with *s_20,w_* of ∼7.6 S, corresponding to a monomer, is seen in the *c(s)* distribution obtained for adalimumab (Fig. 6A). According to the proximity energy theory, in highly concentrated solutions containing mixtures of macromolecules, such as cell lysates or serum, proteins can experience short-range nonspecific attractive interactions leading to the formation of supramolecular assemblies.^55^ Presumably, due to this so-called “preferential solvation” by other serum proteins, the resulting sedimentation coefficient of the adalimumab monomer in serum was slightly larger than that obtained in PBS, confirming the observations of Demeule et al.^24^

When TNF was added to adalimumab, the resulting *c(s)* peaks were not baseline- resolved, which could potentially compromise the accuracy of quantification for individual adalimumab-TNF complexes (Fig. 6A). Nevertheless, the major peaks corresponding to complexes appeared to be at *s_20,w_* values of 11.6 S and 14.8 S, similar to the results obtained in PBS. To evaluate the fractional amount of adalimumab-TNF complexes, the signal originating from the HSA-bilirubin complex as measured in SV experiments performed with human serum alone was subtracted from the total fluorescent signal obtained by integrating the area under the *c(s)* distribution of adalimumab-TNF mixtures. At a 10:1 adalimumab:TNF molar ratio, the adalimumab monomer fraction decreased by more than 10%. Higher concentrations of TNF resulted in a greater decrease, with less than one-third of the adalimumab present in an uncomplexed form at adalimumab:TNF molar ratio of 2:1. Similar to the results obtained in PBS, at equimolar concentrations in serum, no free monomeric adalimumab was present, and approximately a third of the HSA-corrected fluorescence signal corresponded to the 14.8 S peak, whereas higher-order complexes contributed to approximately half of the total signal intensity. In the SV experiments performed with 50 nM and 100 nM adalimumab, at a 2-fold molar excess of TNF over adalimumab, the area under the 11.6 S peak was higher, whereas the amount of higher order complexes was lower (Fig. S3C and S3D). The tendency toward forming smaller complexes when TNF was in molar excess in serum was similar to that obtained in PBS. Even though the peak positions of the major complexes were similar, weight-average *s_20,w_* values from the resulting *c(s)* distributions in human serum, excluding the albumin peak, were larger than those in PBS (Table 2, Table S4). The increase in weight-average *s_20,w_* values could be a consequence of “preferential solvation” of Ada-TNF complexes by serum proteins. Another possibilty is that in serum, several non-specific complexes occur due to weak binding of adalimumab to serum protein components, in addition to Ada-TNF complexes.

The monomer peak in the *c(s)* distribution of 25 nM infliximab in serum was markedly broader than that in PBS and slightly shifted toward larger values, with a *s_20,w_* value of 8.8 S compared with 7.1 S (Fig. 6B). It is difficult to attribute this substantial difference solely to macromolecule-macromolecule preferential solvation, suggesting that binding of serum protein components is most likely responsible for the peak widening. SV data for infliximab-TNF mixtures in serum showed broad continuous sedimentation coefficient distributions, with no discernible complex peaks, contrary to observations in PBS. Furthermore, in contrast to adalimumab, considerably larger amounts of infliximab complexes with sedimentation coefficients above 50 S were detected (Fig. 6B), resulting in higher weight-average *s_20,w_* values with increased TNF concentration (Table 2).

The FDS-SV analysis of etanercept in human serum was complicated by the apparent co-sedimentation of the etanercept and HSA-bilirubin complex, causing an overlap of the corresponding peaks in *c(s)* distributions (Fig. 6C). To estimate the abundance of the etanercept-derived species, the HSA signal measured in serum only was subtracted from the main peak signal. In the absence of TNF, a main peak with *s_20,w_* of 4.4 S corresponding to HSA and etanercept, and a small peak at 7.4 S were observed. Similar to infliximab, the emergence of the 7.4 S-peak, which was absent in the PBS, implies possible interactions between etanercept and serum protein components. In mixtures containing TNF, the main peak corresponding to etanercept-TNF complex was located at 6.5 S and was assumed to arise from the same complex as the 7.0 S-peak detected in PBS, and therefore was attributed to the (Eta)_1_(TNF)_1_ complex. The 8.1 S-peak probably corresponds to Eta-TNF complexes of higher stoichiometry. In contrast to adalimumab and infliximab, at an equimolar ratio approximately one-fifth of the etanercept remained uncomplexed. The peak with the largest sedimnetaion coefficient was located at 10.3 S, indicating that, with the exception of the HSA-related peak, there were no substantial differences in the *c(s)* profiles of etanercept in PBS and human serum.

To further exclude the possibility that fluorescent labeling of antagonists may affect their interaction with TNF, we performed experiments with the same mixing concentrations using 25 nM unlabeled antagonists and 2.5–25 nM labeled TNF (Fig. S7, S8). Within the limitations imposed by the differences in the labeling efficacy, both in PBS and human serum, distributions of antagonists-TNF complexes exhibited essentially similar patterns irrespective of whether fluorescent probe was introduced into antagonist or TNF. The results confirm that labeling does not influence the interaction. In addition, the effects of incubation times on the distribution of the formed complexes were evaluated. Overnight incubation at 37°C caused the loss of fluorescent signal in mixtures formulated in PBS, presumably due to non-specific adsorption of antagonists-TNF to vial surfaces. In the case of human serum, it appears that overnight incubation induces a decrease in the weight-average sedimentation coefficient. This effect is more pronounced in adalimumab than in infliximab mixtures, whereas it is negligible in etanercept mixtures, consistent with the UV-SV results.

### Adalimumab in PBS solutions containing different concentrations of human serum albumin

The distribution of adalimumab and etanercept complexes with TNF was similar in PBS and in human serum. In contrast, a significantly broader distribution, with a considerable amount of larger complexes, was observed for infliximab in human serum compared with PBS (Fig. 5B and 2B). To clarify the mechanism driving differences in complex formation among different TNF antagonists in PBS and human serum, SV experiments were conducted in serial dilutions of HSA in PBS. This approach allowed us to assess the effects of an increasingly crowded environment on antagonist-TNF complex formation in conditions similar to those in human serum, where albumin is the most abundant protein. Because co-sedimentation of HSA with etanercept complicated data interpretation, the experiments were performed using adalimumab.

**Figure 7.**
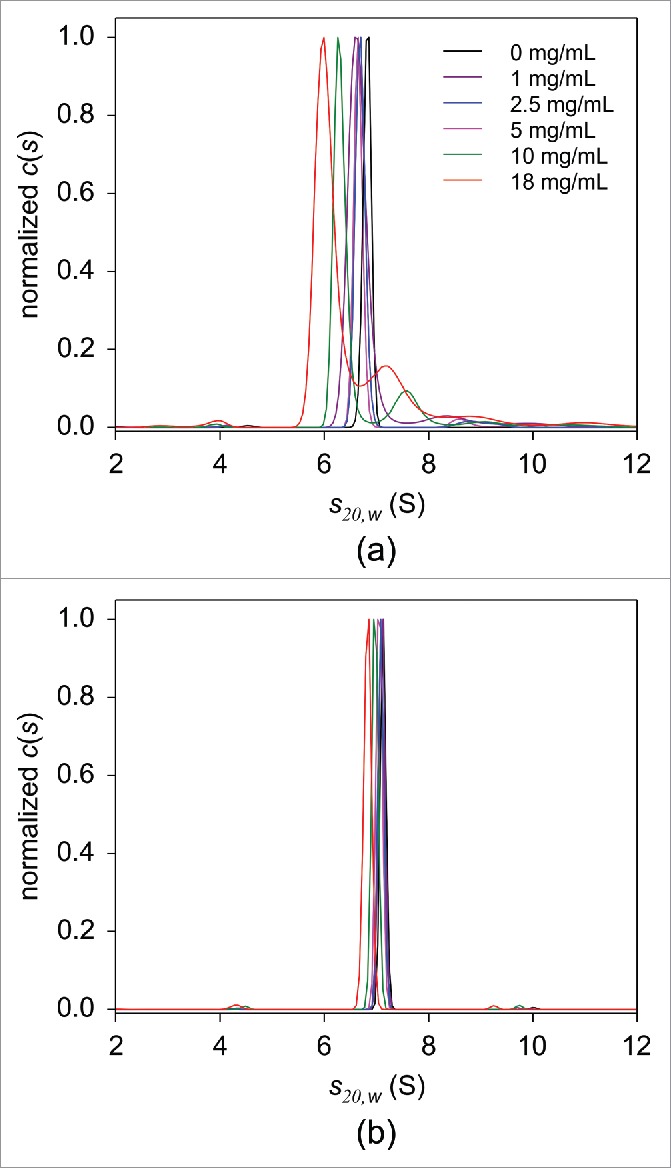
FDS-SV analysis in serial dilutions of HSA in PBS in the absence (a) or presence of 5 mg/mL human immunoglobulin (b). The *c(s)* distributions of 25 nM adalimumab in 0 (black), 1 (purple), 2.5 (blue), 5 (magenta), 10 (green), and 18 mg/mL (red) HSA are shown. For the clarity of presentation, the derived *c(s)* distributions were normalized against the height of the main peak.

As described above, a single peak with a sedimentation coefficient of 6.8 S, corresponding to monomeric adalimumab, was present in the *c(s)* distribution of 25 nM adalimumab in PBS without HSA (Fig. 7A). Upon addition of 1 mg/mL HSA, a peak at 8.4 S was observed (Fig. 7A), presumably resulting from direct interaction between the antibody and serum albumin, consistent with findings recently reported by Hill and Laue.^53^ Increasing amounts of HSA resulted in the overall *c(s_20,w_)* distribution shift toward lower s-values, indicating non-ideal sedimentation behavior. Additionally, an HSA concentration-dependent decrease in the area under the monomer peak, with a concomitant increase in the area under the higher sedimentation coefficient peaks, further support the hypothesis of albumin-immunoglobulin interaction. Thus, at an HSA concentration of ∼18 mg/mL, the monomer peak was located at 6.1 S and comprised less than 70% of the total fluorescence intensity. The possible interaction between adalimumab and HSA did not occur due to non-specific interaction between HSA and the fluorescent probe covalently attached to adalimumab. The area under the peaks, corresponding to putative complexes formed between Alexa-Fluor 488-labeled adalimumab and HSA, decreased with increasing concertation of non-labeled adalimumab (Fig. S9A), indicating that non-labeled adalimumab competes with the labeled adalimumab for binding to HSA, which confirms that their HSA-binding abilities are similar.

Interestingly, adalimumab in human serum (where HSA is present at a concentration of ∼40 mg/mL), other than the bilirubin-HSA peak at 4.1 S, produced a main peak at 7.6 S, which was attributed to monomeric adalimumab, and no larger peaks were detected. To explain the differences in the *c(s_20,w_)* profiles derived from HSA solutions and human serum, we performed SV experiments using HSA dilutions in the presence of human immunoglobulin at a concentration 5 mg/mL. The resulting *c(s_20,w_)* distributions revealed that the observed HSA concentration dependence was considerably diminished with the addition of immunoglobulin. Adalimumab was detected primarily in its monomeric form, indicated by the presence of the 7.0 S peak (Fig. 7B). These results suggest that endogenous serum immunoglobulin interferes with the interaction of adalimumab and HSA.

In contrast to the *c(s_20,w_)* profiles derived from SV experiments in PBS, where the major Ada-TNF peaks at 11.9 S and 14.5 S were well-resolved (Fig. 2A), in PBS containing 40 mg/mL HSA, broad distributions of complexes with no discernable peaks were obtained for the mixtures of 25 nM adalimumab and 0–25 nM TNF (Fig. S9B). Despite these differences, the weight-average sedimentation coefficient decreased with increasing TNF concentration irrespective of whether HSA was present (Table S5).

### Measurement of FcγR activation by antagonist-TNF complexes

**Figure 8.**
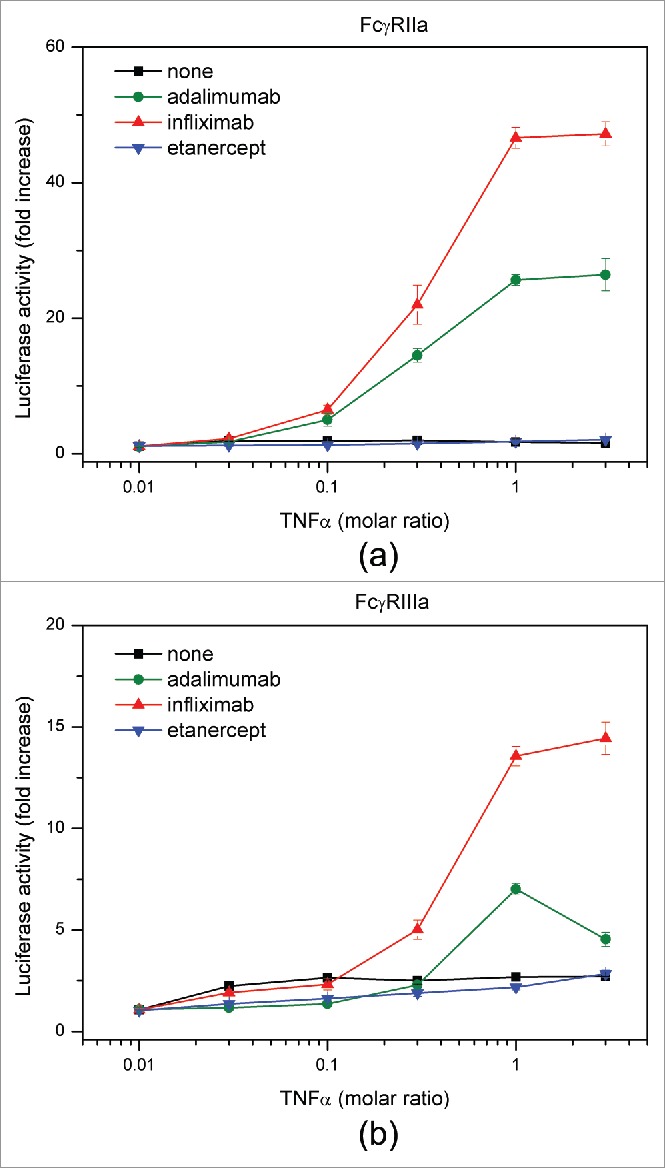
FcγR reporter cell assay. Activation of FcγRIIa (a) and FcγRIIIa (b) by adalimumab (green), infliximab (red), and etanercept (blue) in the presence of TNF was measured.

We evaluated the ability of antagonists to bind FcγRs to elucidate the effects of different complex sizes among the 3 antagonists on FcγR-induced activation of immune cells. In the absence of TNF, antagonists exhibited the same degree of FcγR binding (data not shown). We performed a cell-based reporter assay using Jurkat/FcγRIIa/NFAT-Luc and Jurkat/FcγRIIIa/NFAT-Luc cells.^56^ Infliximab-TNF and adalimumab-TNF complexes effectively activated both cell lines in a TNF concentration-dependent manner, and the activity of infliximab was considerably higher than that of adalimumab (Fig. 8). In contrast, etanercept-TNF complexes caused no change in the luciferase activity of reporter cells. These results suggest that the extent of FcγR activation depends on the size of antagonist-TNF complex.

## Discussion

We performed biophysical characterization of TNF-antagonist complexes for 3 antagonists, adalimumab, infliximab, and etanercept, comparing complexes formed in human serum with those formed in PBS using AUC FDS-SV. We demonstrated that complex size varies among the 3 antagonists, providing a rationale for differences in the induction of downstream signaling pathways via antagonist complex interaction with FcγRs.

In the absence of TNF, adalimumab sedimented as a monomer in both PBS and human serum with *s_20,w_* values of 6.8 S and 7.5 S, respectively. Weight-average sedimentation coefficients calculated for solutions containing adalimumab and TNF at the same concentration were higher in human serum than in PBS (Table 2). The apparent increase in protein sedimentation coefficients in highly concentrated solutions due to a preferential solvation by other macromolecules has been discussed previously.^55^ However, our data suggest that the increased sedimentation coefficient of adalimumab in human serum instead results from interaction with the serum protein components. In PBS, the area under peaks with higher sedimentation coefficient than adalimumab monomer was larger with higher concentrations of HSA, indicating a possible interaction between adalimumab and HSA (Fig. 7A).

Despite differences in the weight-average sedimentation coefficients, the *c(s_20,w_)* distributions in PBS and human serum exhibited similar features. A relatively broad distribution of adalimumab-TNF complexes, with the major peaks located at ∼11.9 S and 14.5 S, was seen. Based on the results of SV data analysis using the hybrid local continuous distribution and global discrete species model of SEDPHAT, these peaks were attributed to (Ada)_2_(TNF)_1_ and (Ada)_3_(TNF)_1_ complexes, respectively (Table 1). In addition, (Ada)_1_(TNF)_1_ and (Ada)_3_(TNF)_2_ complexes were identified.

Similar findings regarding the wide distribution pattern of adalimumab-TNF complexes have been previously reported. Data obtained using SEC revealed that adalimumab:TNF complexes span a wide range of molecular weights: 600–5,800 kDa^20^ or up to 4,000 kDa.^21^ The molecular weight of the most thermodynamically stable complex was determined to be ∼598 kDa. Therefore, it was concluded that the complex was composed of 3 adalimumab molecules and 3 TNF molecules.^20^ However, our SV (Fig. 2A), MS (Fig. 3), and simulation analysis results (Fig. 5) do not support this stoichiometry, but rather suggest a relationship between complex size and adalimumab-TNF concentration and molar ratio. At equimolar concentration, or with TNF in excess over adalimumab, a smaller peak with *s_20,w_* of 8.2 S, which was attributed to an (Ada)_1_(TNF)_1_ complex, was revealed (Fig. 2A, Fig. S3A). Data in Table 2 show that the weight-average sedimentation coefficient is initially higher with a higher relative adalimumab concentration, and then lower at an equimolar mixing ratio. SV experiments performed with 50 nM and 100 nM adalimumab confirmed that samples containing TNF at greater than equimolar mixing ratios exhibited lower weight-average sedimentation coefficients than those below the equimolar ratio (Table S4). Furthermore, simulation data displayed a similar trend toward increased abundance of complexes of lower stoichiometry with higher TNF concentrations (Fig. 5). Thus, when adalimumab is in excess, the trimeric molecules of TNF are bound by more than 2 molcules of adalimumab, whereas when TNF is in excess, (Ada)_1_(TNF)_1_ complexes are detected with a corresponding decrease in the abundance of complexes with higher stoichiometries.

Native MS revealed the presence of adalimumab-TNF complexes with 1:1, 2:1, 2:2, and 3:2 stoichiometries, consistent with the AUC results (Table 1). However, the (Ada)_3_(TNF)_1_ complex to which the 14.5 S-peak in the *c(s)* distribution was assigned was absent. Instead, a 408,047 Da band corresponding to (Ada)_2_(TNF)_2_ was detected. As discussed above, the slight difference in complexes detected by AUC and MS is likely attributable to differences in concentration and molar ratios at which measurements were performed.

The importance of determining the binding profiles of small molecule drug candidates to serum protein components, and incorporating these analyses into drug development protocols has been emphasized.^57,58^ Interactions with plasma proteins can have a substantial effect on the drug pharmacokinetic properties and reduce its clinical efficacy.^59^ Conversely, the circulatory half-lives of proteins, peptide-based drugs, and small molecules can be considerably extended by association with endogenous serum albumin.^60,61^ The neonatal Fc receptor (FcRn) plays an important role in this process by binding to drug-carrying albumin in the same way it binds to albumin and IgG, preventing their degradation via cellular recycling pathways.^62^ The application of FDS-SV appears to be particularly useful to elucidate possible interactions of target biopharmaceuticals with serum protein components at physiologically relevant concentrations, which cannot be accounted for using other analytical techniques.

No separate peaks could be discerned in the sedimentation coefficient distribution pattern of adalimumab-TNF complexes in PBS solutions containing 40 mg/mL HSA, and it was considerably broader than that in the absence of HSA (Fig. S9). Interestingly, even though the *c(s)* distribution in human serum was wider than that in PBS, it was narrower than in PBS containing HSA. These observations imply that HSA may bind weakly to adalimumab-TNF complexes when present at over 1000-fold molar excess, whereas in human serum this low affinity interaction is considerably reduced. In SV experiments with adalimumab in HSA solutions, the formation of putative adalimumab-HSA complexes was abolished in the presence of human immunoglobulin. We therefore suggest that endogenous immunoglobulin in human serum is primarily responsible for the diminished adalimumab-HSA interaction.

The formation of complexes between fluorescently labeled protein OPG at picomolar concentrations and serum albumin as low as 1 mg/mL has been recently observed.^53^ Analogous to our results, the peak corresponding to the HSA-OPG complex in HSA solutions was not detected in serum, and it was concluded that endogenous compounds in the serum compete with the OPG molecules for binding to HSA. Our data suggest that this component might be immunoglobulin, which is abundantly present in human serum. However, to our knowledge, no data showing an interaction between immunoglobulin and human serum albumin are available, and further studies are required to assess the mutual influence of endogenous proteins on binding to serum albumin.

The *c(s)* distribution of infliximab-TNF complexes in PBS was similar to adalimumab with regard to complex stoichiometry, but with larger weight-average sedimentation coefficients (Tables 1, 2). Our data clearly demonstrated that the complexes that are formed and their fractional abundances depended on mixing ratios (Fig. 2B). In contrast to results previously reported by Scallon et al.,^23^ where a single (Inf)_3_(TNF)_1_ complex was detected when TNF was in molar excess over infliximab, the additional formation of complexes with lower stoichiometries was observed in our study. The *c(s)* profile of infliximab-TNF complexes in human serum was markedly different than in PBS, showing a continuous distribution of complexes with no distinct peaks (Fig. 6B). The weight-average sedimentation coefficient of infliximab complexes at each TNF molar ratio was higher than that of adalimumab (Table 2).

In contrast to adalimumab and infliximab, etanercept formed exclusively 1:1 complexes with TNF in both PBS and human serum, with only a small amount of complexes with higher binding stoichiometry detected. This finding is consistent with the previous studies where etanercept formed 1:1 complexes.^21-23^ Overall, our data showed clear differences in the sizes of complexes formed between these 3 antagonists and TNF. Both in PBS and in human serum, the largest complexes were formed by infliximab, followed by adalimumab, whereas the smallest complexes were formed by TNF and etanercept.

Different clinical efficacy and safety profiles of the 3 different TNF antagonists have been reported, though the mechanisms responsible for these differences remain to be elucidated. A number of clinical investigations have studied the use of these TNF inhibitors, and they suggest that adalimumab has an advantage in therapeutic treatment.^63^ Nevertheless, the incidence of ADA in psoriasis patients being treated with infliximab and adalimumab was similar.^13^

The differences between TNF antagonists' therapeutic actions have been partly addressed in crystallographic studies. The 3-dimensional structures of infliximab-Fab-TNF^64^ and adalimumab-Fab-TNF^63^ complexes have been determined, and, even though it was shown that the adalimumab epitope occupies a larger area compared with infliximab, both epitopes overlap with the TNFR binding site in a similar manner. The complexes consisted of one TNF trimer bound by the 3 Fab molecules, in agreement with our AUC results (Table 1). However, the reported structural data are insufficient to explain the ability of infliximab to form larger complexes with TNF compared with adalimumab, as revealed in our study.

Because etanercept is a soluble TNFR2-Fc recombinant protein, its epitope on TNF is also located within the TNF-receptor binding site. Although 3 molcules of TNFR2 can potentially bind to a single trimeric TNF,^65^ in etanercept, due to the structural arrangement and spatial hindrance, where the TNFR2 portion of etanercept contacts with 2 adjacent TNF protomers and the Fc portion fused to TNFR2, the remaining binding site on TNF is considered to be inaccessible to the TNFR2 portion of other etanercept molecules.^63^ Therefore, it was concluded that one etanercept molecule binds to one trimeric TNF molecule, which is consistent with our AUC data.

In the pathogenesis of autoimmune disorders, immune complexes have been implicated in the triggering of inflammatory responses via activation of the complement pathway or through interaction with activating FcγRs expressed on immune cells.^66^ It was suggested that larger complexes may be more effective in activating complement, leading to faster clearance of TNF from circulation. Likewise, it has been suggested that larger complexes can be cleared faster than smaller complexes via FcγRs-mediated activation of immune cells.^67^

In an attempt to gain a better understanding of the possible correlation between anti-TNF drug safety and efficacy and sizes of the respective complexes with TNF, activation of FcγRs by adalimumab, infliximab, and etanercept in the presence of TNF was evaluated by an FcγR reporter cell assay. The results demonstrated the highest FcγR-mediated activation of the downstream immune signaling pathway occurred with infliximab, followed by adalimumab, whereas no significant increase in activation of FcγR could be observed for etanercept (Fig. 8). We suggest that enhancement of the FcγR-mediated signal transduction can be attributed to a size-dependent increase in binding of mAb-TNF complexes to FcγRs, which results in more efficient clustering of the receptors. It has been shown that in the absence of exogenous TNF, adalimumab, infliximab, and etanercept were only capable of low-level binding to the activating receptors FcγRIIa and FcγRIIIa. Upon addition of TNF, however, increased binding of mAbs, but not etanercept was observed, which was suggested to be mainly due to the formation of large protein complexes by the mAbs. Consistent with these in vitro observations, adalimumab and infliximab, but not etanercept, induced antibody-dependent cell-meditated cytotoxicity.^68^

Increased activation of FcγR leads to faster clearance of both antigens and antibodies by enhanced uptake and breakdown of the complexes. Immune cells that express activating FcγR, such as monocytes and macrophages, produce proinflammatory cytokines, particularly interferon-γ, which induces the expression of class II MHC that can, in turn, increase the presentation of the fragments of drug antibody and stimulate ADA generation. Thus, the shorter serum half-life of infliximab, as well as the higher incidence of ADA formation compared with adalimumab, can be explained by the formation of larger complexes, although further studies may be needed to increase the generalizability of these findings.

Self-aggregation is one of the major concerns with regard to the clinical safety of biological therapeutics.^69,70^ Because the aggregated forms of mAbs resemble immune complexes, they may play an important role in inducing ADA formation.^71^ Correlation between sizes of aggregates and immunogenicity have been considered, and larger micron-sized aggregates, often referred to as sub-visible particles, were suggested to have a higher propensity to induce immune response than nanosized aggregates.^72^ Using the Stokes-Einstein relationship, we estimated the hydrodynamic radii of the peaks present in the *c(s)* distribution of adalimumab and infliximab in human serum and concluded that the soluble antagonist-TNF complexes were smaller than 100 nm. Nevertheless, our cell-reporter assay clearly showed that these complexes were able to induce considerable FcγR activation, suggesting a potential role in immunogenicity and manifestation of adverse effects.

In conclusion, the results reported here provide clear evidence that FDS-SV is an extremely valuable technique for the prediction of therapeutic action and in-depth understanding of the binding of target protein drugs to their respective antigens directly in human serum at physiologically relevant concentrations. It is anticipated that incorporating FDS-AUC in the drug development process will positively contribute to optimal design of pharmaceuticals with enhanced efficacy and minimized immunogenicity.

## Materials and methods

### Proteins

Adalimumab (Humira®) was purchased from Eisai, infliximab (Remicade®) was obtained from Mitsubishi Tanabe Pharma, and etanercept (Enbrel®) was received from Takeda Pharmaceutical Company Limited. Recombinant human soluble TNF was produced recombinantly using a silkworm baculovirus expression system by ProCube® Biotechnology Center at the Sysmex Research and Development Center in Kobe. Recombinant human TNF purified from *E coli* was purchased from R&D Systems Inc. FDS-SV experiments performed with adalimumab and TNF prepared using a baculovirus and an *E coli* expression system yielded essentially the same results (Fig. S3 and S10) and therefore, unless otherwise stated, baculovirus-expressed TNF was used in all experiments.

### Methods

#### Absorbance-detected sedimentation velocity analytical ultracentrifugation

Two μM respective antagonist and 2 μM TNF were mixed in PBS pH 7.4 (Gibco) and incubated at 20°C for 2 hours or at 37°C overnight before AUC. Sedimentation velocity (SV) experiments with the resulting mixtures and with 2 μM antagonists in the absence of TNF were performed using an Optima XL-I analytical ultracentrifuge equipped with absorbance optics (Beckman Coulter) at rotor speed of 42,000 rpm. Scans were acquired at a wavelength of 280 nm with 30-μm radial increments. Experiments with 2 μM TNF were conducted at a rotor speed of 60,000 rpm using absorbance detection at 230 nm.

#### Dynamic light scattering

DLS was conducted at 25°C using ZetaSizer Nano (Malvern). Adalimumab, infliximab, and etanercept were diluted to 3.3 μM using PBS, filtered by 0.22 μm-pore size syringe filter (Millipore), and mixtures containing the respective antagonist:TNF at molar ratios of 1:0, 1:0.3, and 1:1 were prepared.

#### Protein labeling

Labeling of target proteins with a fluorescent probe that can be excited using 488-nm laser light and which emission wavelengths are greater than 505 nm is necessary for FDS measurements. We chose Alexa Fluor® 488 fluorescent dye (Molecular Probes®), which meets these requirements, and is also relatively insensitive to pH, and highly resistant to photobleaching. In addition, it was shown to be stable during AUC experiments conducted in serum.^24,53^ Labeling of adalimumab, infliximab, etanercept, and TNF was performed according to the following protocol. Each TNF antagonist was at first diluted to 15 mg/mL to a final volume of 100 μL. Then, 10 μL of 1 M sodium bicarbonate (pH 8.3) and 2.2 μL of 10 mg/mL Alexa Fluor® 488 stock solution (5 mg reactive dye/0.5 mL dimethyl formamide) were added and the resulting mixture was allowed to react for 4 h at room temperature. The reaction was quenched with 10 μL of 1.5 M hydroxylamine (pH 8.5) and mixture was allowed to react for another 1 h at room temperature. The purification of Alexa-labeled antagonists was performed by SEC using Alliance 1100 HPLC system with TSKG3000 SWXL column and 1× PBS pH 7.4 as the mobile phase. The final solution concentrations were 1.8, 1.4, 2.5, and 1.2 μM for adalimumab, infliximab, etanercept, and TNF, respectively.

#### Fluorescence-detected sedimentation velocity analytical ultracentrifugation

Twenty-five nM respective labeled antagonist and 0–25 nM TNF were mixed in PBS or human serum (MP Biomedicals) and incubated at 20°C for 2 hours before FDS-SV AUC. Similar mixtures containing 25 nM unlabeled antagonist and 2.5–25 nM labeled TNF were incubated at 20°C for 2 hours or at 37°C overnight before FDS-SV AUC. Additionally, mixtures containing 50 nM and 100 nM labeled adalimumab were analyzed. The experiments were performed using fluorescence optics (Aviv Biomedical) at a rotor speed of 42,000 rpm. To prevent non-specific adsorption of protein to centerpiece walls and windows, 0.1 mg/mL lysozyme was added to PBS.^34^ To clarify the mechanism driving differences in complex formation among different TNF antagonists in PBS and human serum, SV experiments were conducted in serial dilutions of pooled human serum albumin in PBS.

The acquired data were analyzed with SEDFIT (version 14.6e) using the continuous *c(s)* distribution model.^39,73^ The apparent sedimentation coefficients were converted to *s_20,w_* using density and viscosity of the respective buffer solutions measured using an Anton Paar density meter DMA4500 and viscometer Lovis 2000ME, respectively. To evaluate the fractional amount of antagonist-TNF complexes, the signal originating from the HSA-bilirubin complex as measured in SV experiments performed with human serum alone was subtracted from the total fluorescent signal obtained by integrating the area under the *c(s)* distribution of antagonist-TNF mixtures.

#### Mass spectrometry under non-denaturing conditions

TNF, adalimumab, and infliximab were buffer-exchanged into 150 mM ammonium acetate, pH 6.8 by passing the proteins through a Bio-Spin 6 column (Bio-Rad). The buffer-exchanged TNF (2 μM trimer) and respective antagonist (2, 2.5, 3, and 4 μM) were mixed, incubated at 25°C for 20 min, and analyzed by nanoflow electrospray ionization MS using gold-coated glass capillaries made in-house (∼2–5 µL sample loaded per analysis). Spectra were recorded on a SYNAPT G2-S*i* HDMS mass spectrometer (Waters Corporation) in positive ionization mode at 1.33 kV with a 150 V sampling cone voltage and source offset voltage, 0 V trap and transfer collision energy, and 5 mL/min trap gas flow. The spectra were calibrated using 1 mg/mL cesium iodide and analyzed using MassLynx software (Waters Corporation).

#### Isothermal titration calorimetry

ITC experiments were performed with a Fab fragment and full-length adalimumab at 25°C using an iTC200 (GE Healthcare) or PEAQ-ITC (Malvern) instruments. Prior to each experiment, TNF, adalimumab-Fab, and full-length adalimumab were extensively dialyzed against PBS containing 0.1% polysorbate 80. For adalimumab-Fab binding to TNF, 9.8 μM adalimumab-Fab was placed into the cell and 39.8 μM trimeric TNF was loaded into the syringe. For the experiments with full-length adalimumab, 1.7 μM adalimumab was placed into the cell and the 27.5 μM TNF was loaded into the syringe. Titrations consisted of a preliminary 1-μL injection, followed by 2-μL injections with a 120 s interval between 2 successive injections. The integration of thermograms was performed using NITPIC software^74^ and the resulting isotherms were fit to a A+B+B+B AB+B+B ⇌ ABB+B ⇌ ABBB binding model using the SEDPHAT software.

#### FcγR reporter assay

Stable cell lines Jurkat/FcγRIIa/NFAT-Luc and Jurkat/FcγRIIIa/NFAT-Luc expressing FcγRIIa or FcγRIIIa, respectively, were established as described in.ref. ^56^ TNF antagonists were mixed with TNF at different molar ratio and incubated for 30 mins at 37°C. Mixtures of respective antagonist and TNF were added to Jurkat/FcγRs/NFAT-Luc cells to the final concentration of antagonist of 1 μg/mL. After incubation for 4 hr at 37°C in 5% CO_2_, luciferase activities were measured by using ONE-Glo Luciferase Assay System (Promega).

## Supplementary Material

Supplemental_Material.docx
