# Supplementary material for: Analytical ultracentrifugation with fluorescence detection system reveals differences in complex formation between recombinant human TNF and different biological TNF antagonists in various environments
Source: MAbs. 2017 Mar 3;9(4):664–79. doi: 10.1080/19420862.2017.1297909 (PMC5419078; doi:10.1080/19420862.2017.1297909)
Supplement: Supplemental_Material.docx [file kmab-09-04-1297909-s001.docx]

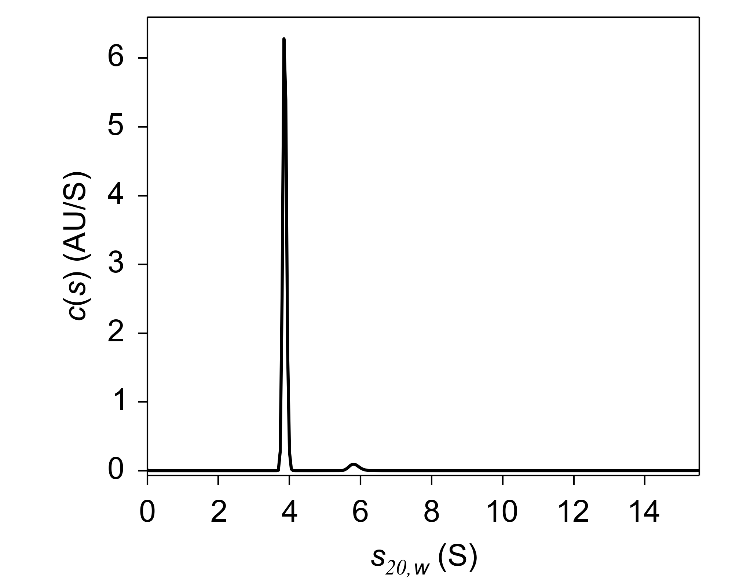
**
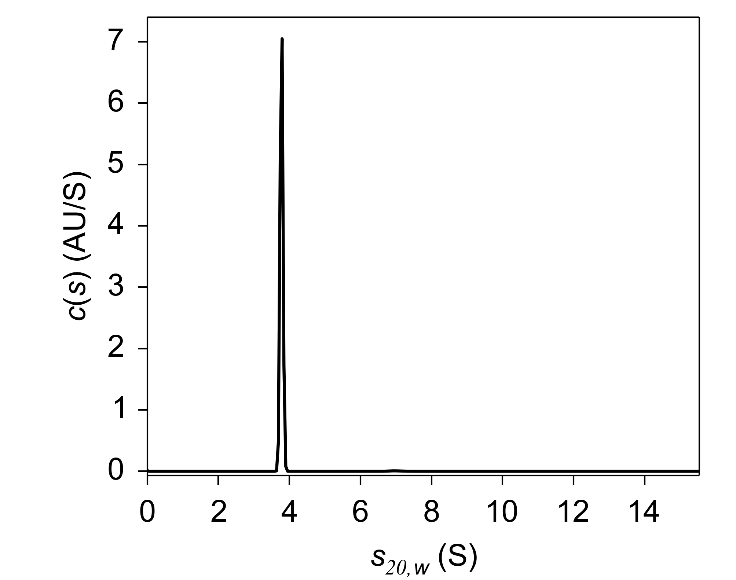
**

**A** **B**


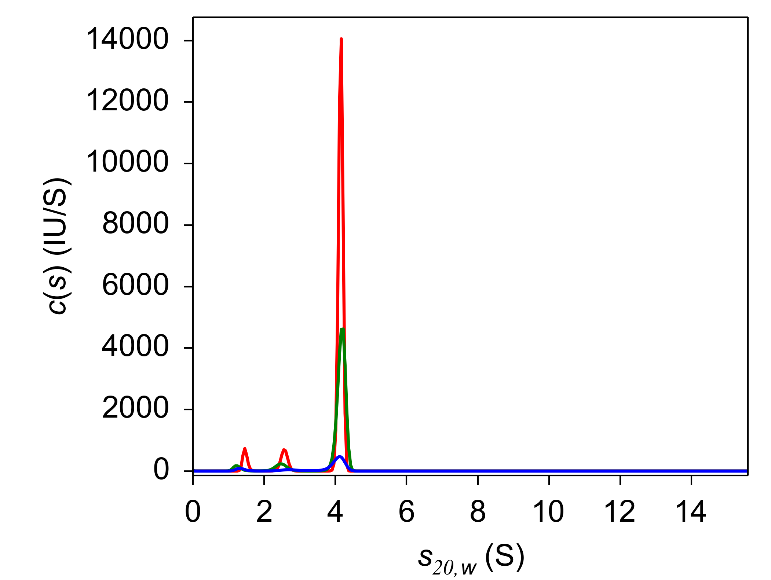

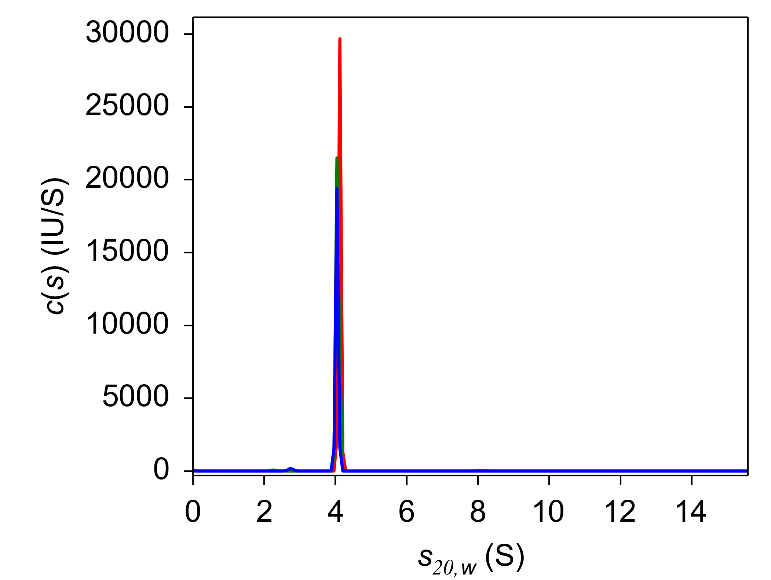


**Supplementary figure 1.** Determination of oligomeric states of human TNF. Sedimentation coefficient distributions for 2 μM recombinant human TNF produced using a baculovirus expression system (**A**) and an E. coli expression system (**B**) obtained using UV-SV are shown. Sedimentation coefficient distributions for 2.5 (blue), 12.5 (green), and 25 nM (red) Alexa-labeled baculovirus-expressed human TNF obtained in PBS (**C**) and human serum (**D**) using FDS-SV are shown. In human serum, co-sedimentation of TNF and HSA-bilirubin complex caused an overlap of the corresponding peaks and therefore a single peak with sedimentation coefficient s_20,w_ of approximately 4.1 S was observed in the resulting c(s) distributions.

**C** **D**

**

Supplementary figure 2.** DLS analysis of the TNF antagonists in the presence of various amounts of TNF. Particles size distributions in the solutions of adalimumab (bottom panel), infliximab (middle panel), and etanercept (top panel) in the absence of TNF (black) and in the mixtures containing the respective antagonist:TNF at molar ratios of 3:1 (blue) and 1:1 (red) are shown.

A B


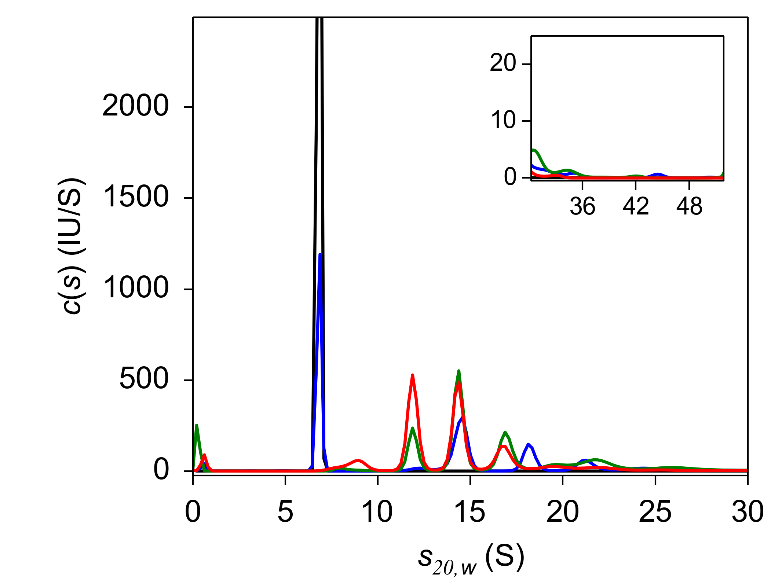

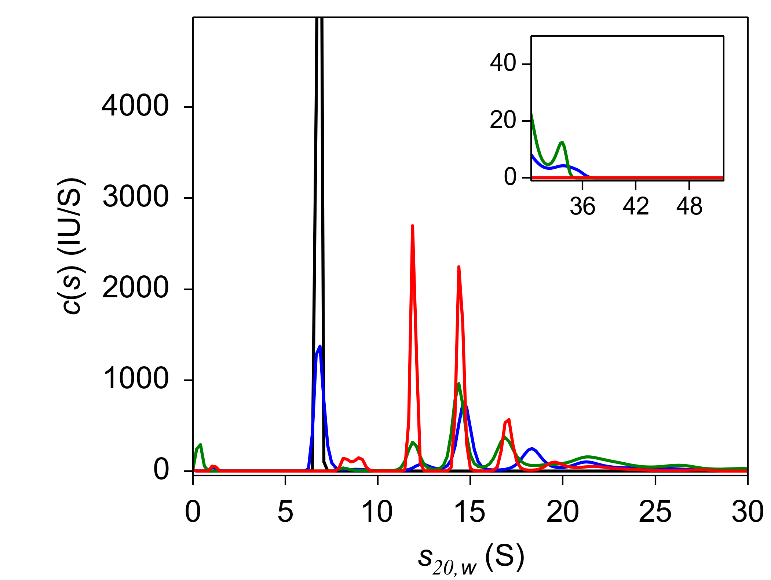

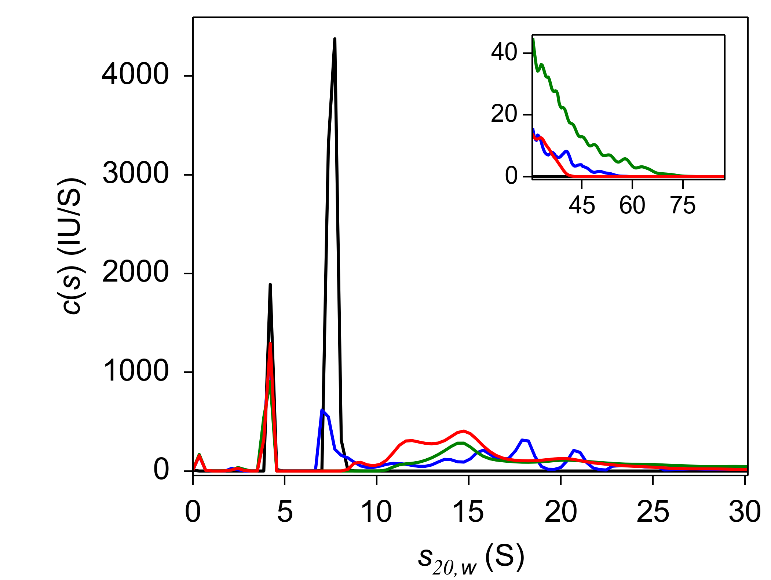

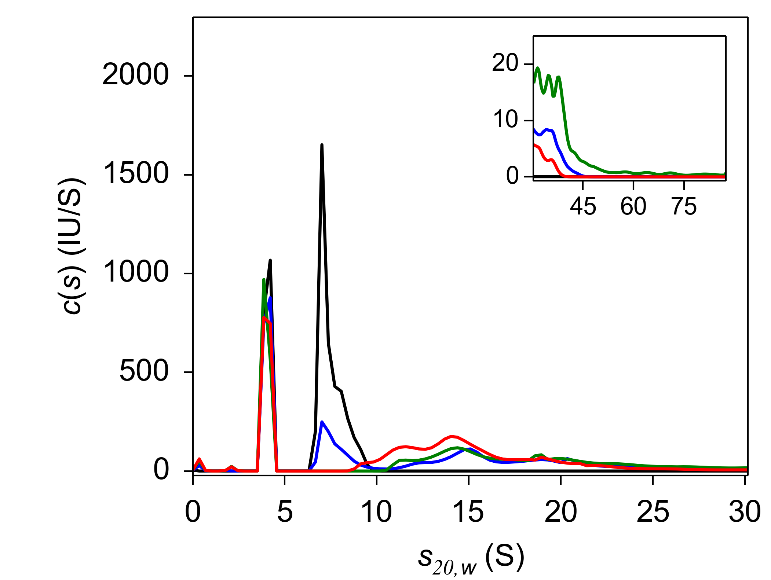


**Supplementary figure 3.** FDS-SV with 50 nM and 100 nM adalimumab in the presence of varying concentrations of TNF produced using a baculovirus expression system. (**A**) 50 nM adalimumab in PBS; (**B**) 100 nM adalimumab in PBS; (**C**) 50 nM adalimumab in human serum; (**D**) 100 nM adalimumab in human serum The c(s) distributions in the absence of TNF (black) and of the 2:1 (blue), 1:1 (green), and 1:2 (red) molar mixtures of adalimumab:TNF are shown.

C D


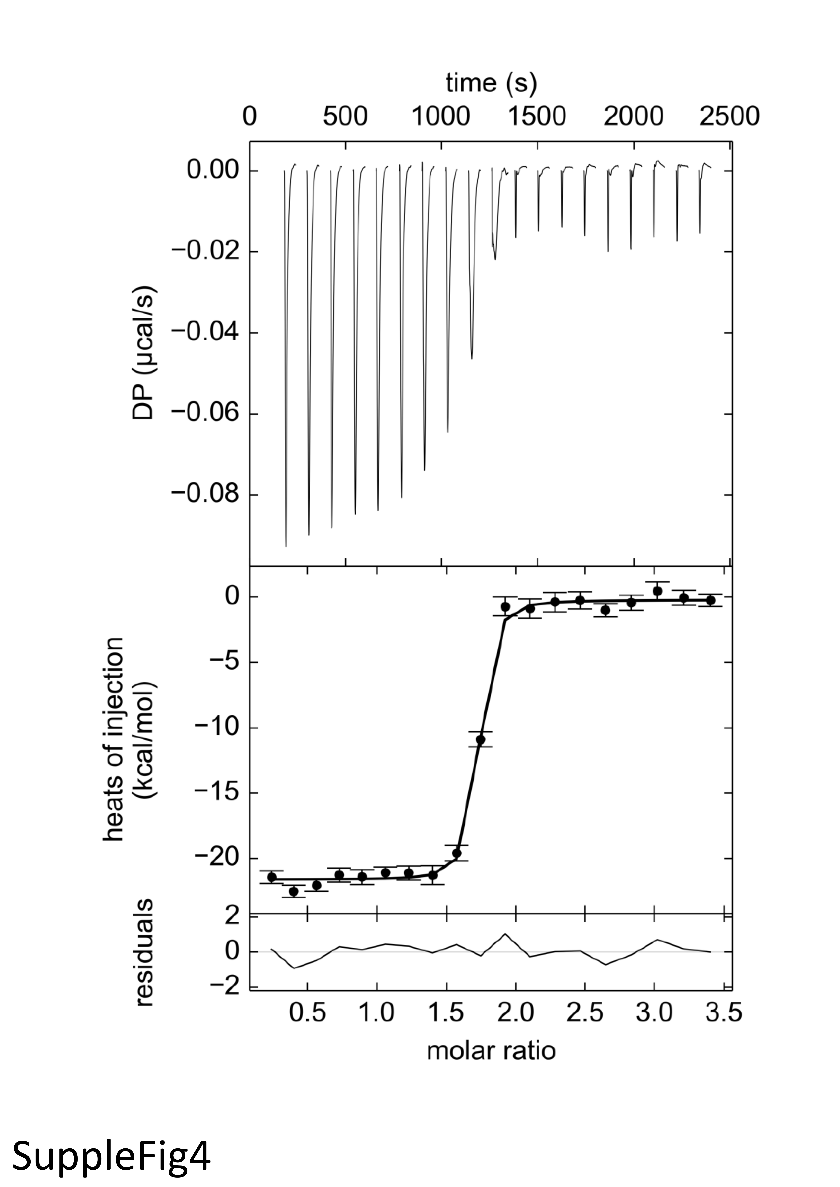


**Supplementary figure 4**. ITC analysis of direct titration of 27.5 µM trimeric TNF into 1.7 µM full-length adalimumab. Raw heat changes (top panel), normalized heat changes with best-fit values (solid line) (middle panel), and residuals of the fit (bottom panel) are shown. Non-linear regression analysis of the curves using A + B <-> AB binding model yielded the Kd and ΔH values of 2.2 nM and -21.4 kcal/mol, respectively.

**Supplementary figure 5.** Mass spectra of the TNF-infliximab complexes. Mass spectra of the mixtures containing trimeric TNF:infliximab at molar ratios of 1:0 (*black*), 1:1 (*orange*), 1:1.25 (*green*), 1:1.5 (*red*), 1:2 (*cyan*), and 0:1.5 (*purple*) are shown. Inset shows expansion of the spectrum of the 1:1.5 trimeric TNF:infliximab mixture.

**A** **B**


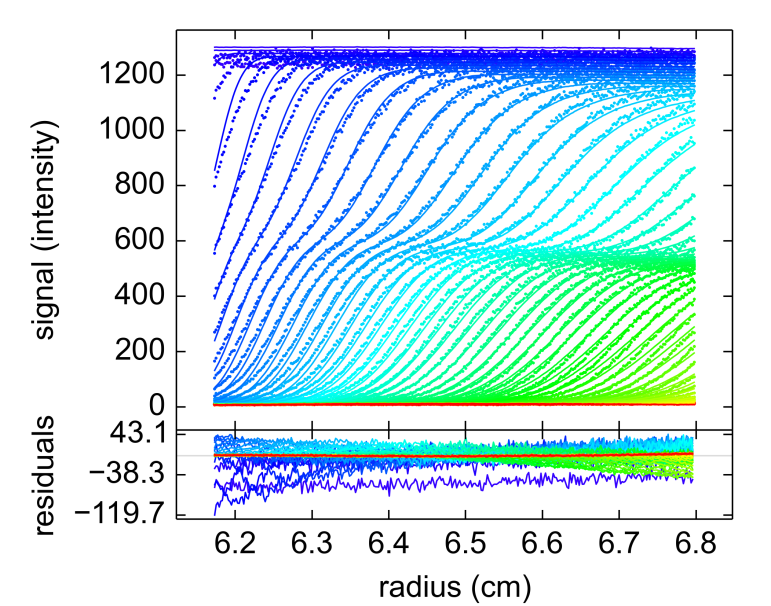

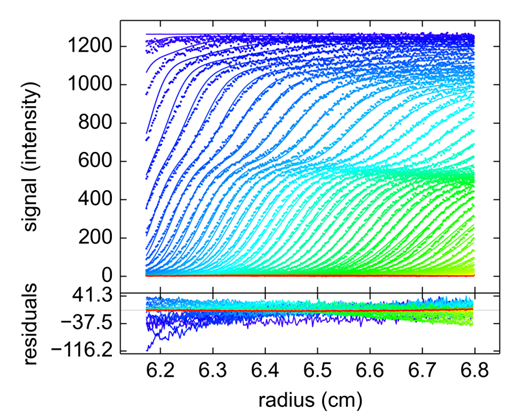

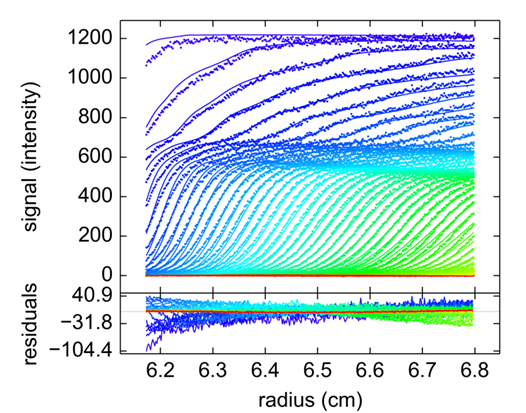

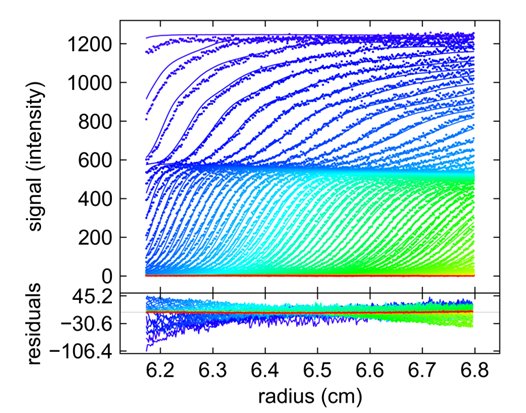


**Supplementary figure 6.** Representative raw FDS-SV data acquired in human serum shown together with the fitted curves. The data of 25 nM adalimumab in the absence of TNF (**A**) and of the mixtures containing trimeric TNF:adalimumab at molar ratios of 1:10 (**B**), 1:2 (**C**), and 1:1 (**D**) are shown.

**C** **D**


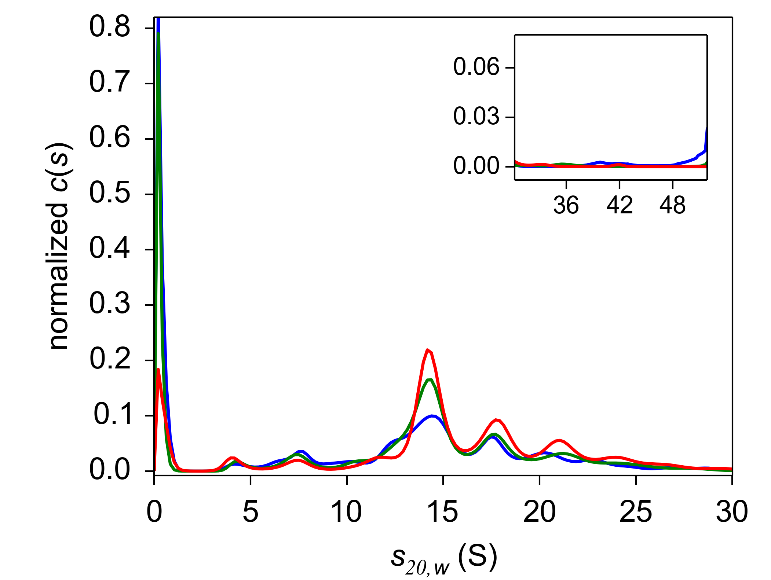

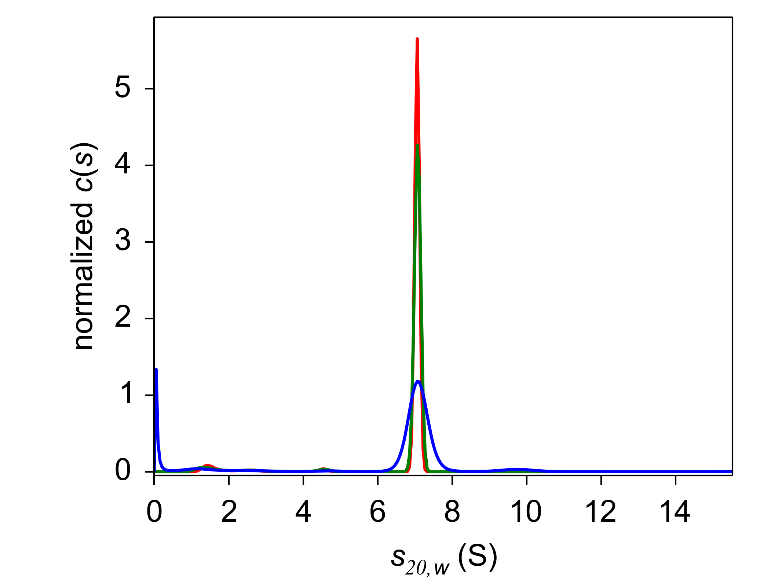


**Supplementary figure 7.** FDS-SV with 25 nM unlabeled adalimumab (**A**), infliximab (**B**), and etanercept (**C**) in the presence of varying concentrations of labeled TNF in PBS. The c(s) distributions of the 10:1 (blue), 2:1 (green), and 1:1 (red) molar mixtures of the respective antagonist:TNF are shown. The distributions were normalized against the area.


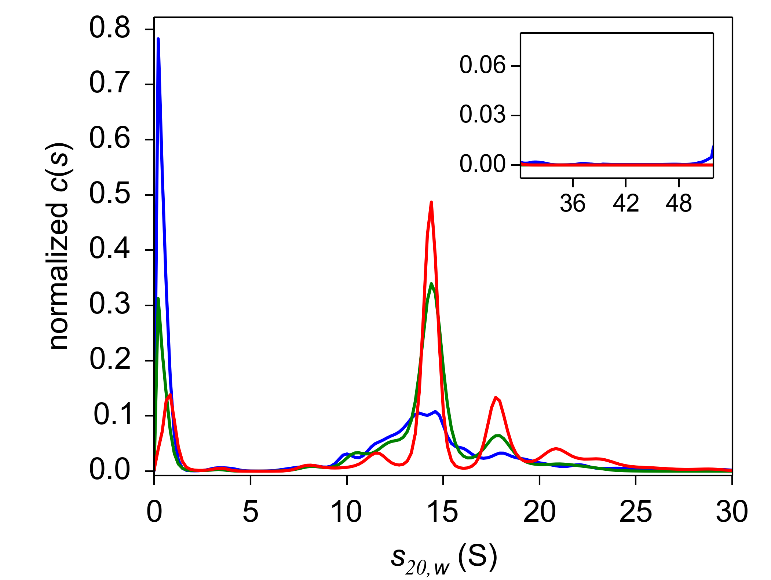


**A** **B**

**C**

**A** **B**


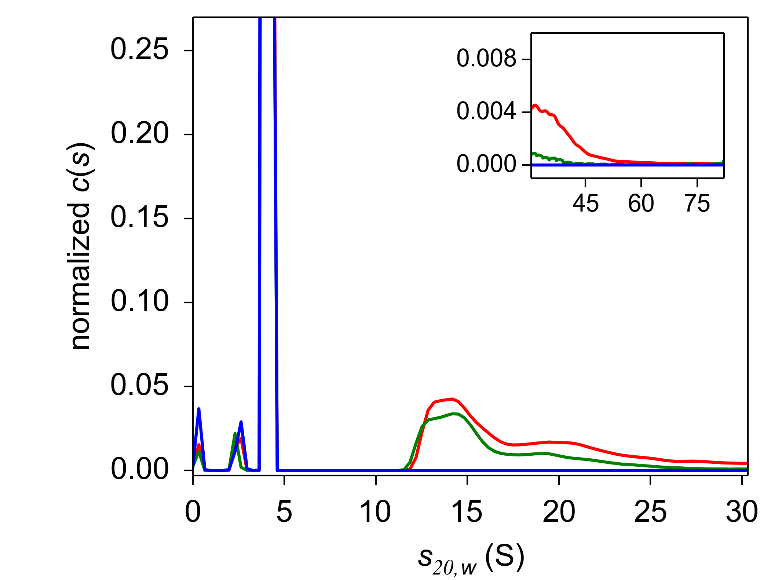

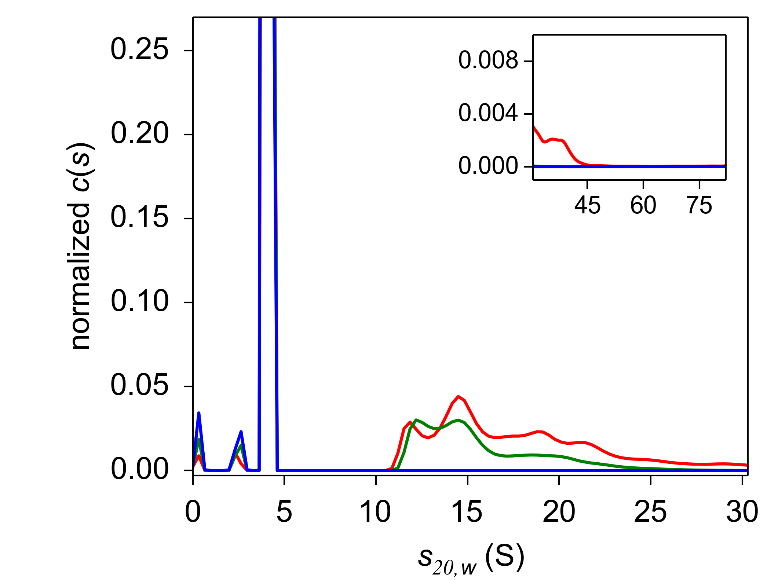

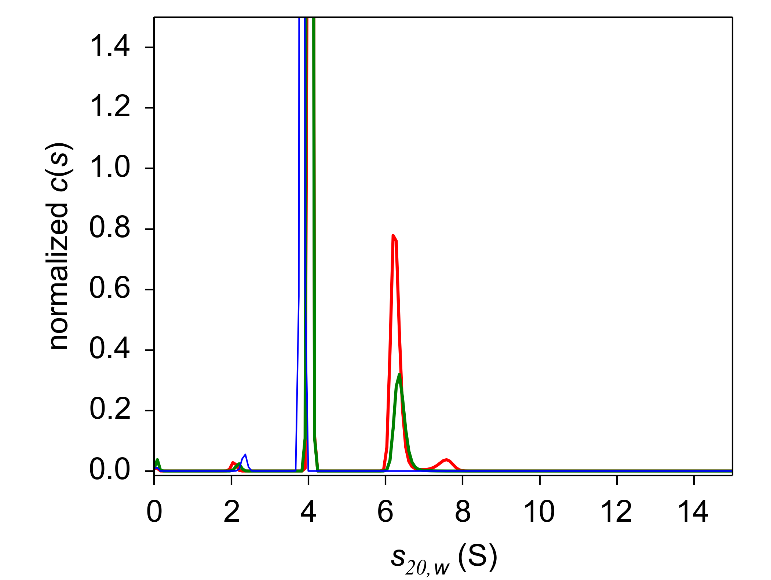


**Supplementary figure 8.** FDS-SV with 25 nM unlabeled adalimumab (**A**), infliximab (**B**), and etanercept (**C**) in the presence of varying concentrations of labeled TNF in human serum. The c(s) distributions of the 10:1 (blue), 2:1 (green), and 1:1 (red) molar mixtures of the respective antagonist:TNF are shown. The distributions were normalized against the area.

**C**


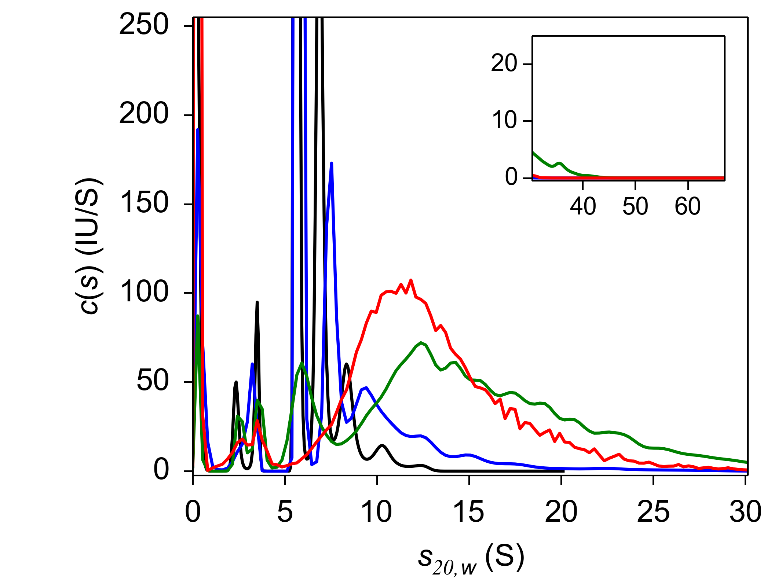

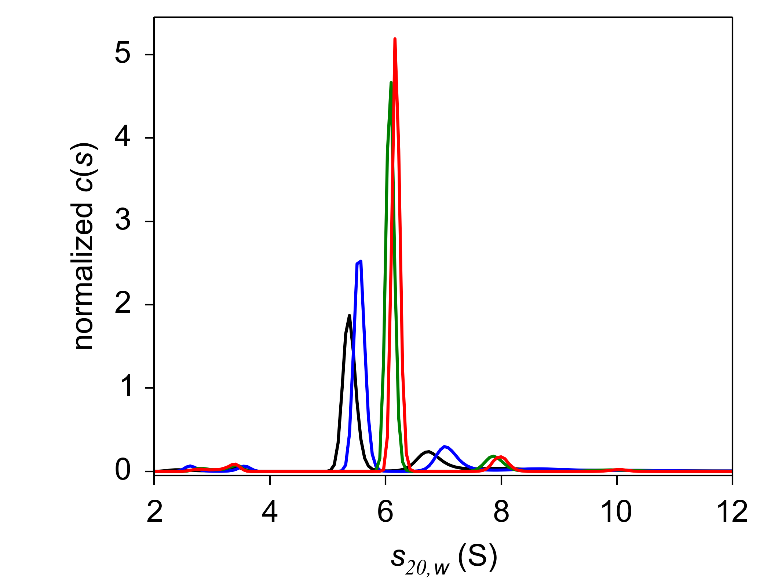


**Supplementary figure 9.** FDS-SV with 25 nM adalimumab in PBS solutions containing approximately 40 mg/mL HSA. (**A**) The c(s) distributions in the absence of unlabeled adalimumab (black) and of the 1:1 (blue), 1:2 (green), and 1:4 (red) molar mixtures of labeled:unlabeled adalimumab. The distributions were normalized against the area. (**B**) The c(s) distributions in the absence of TNF (black) and of the 10:1 (blue), 2:1 (green), and 1:1 (red) molar mixtures of adalimumab:TNF.

**A** **B**


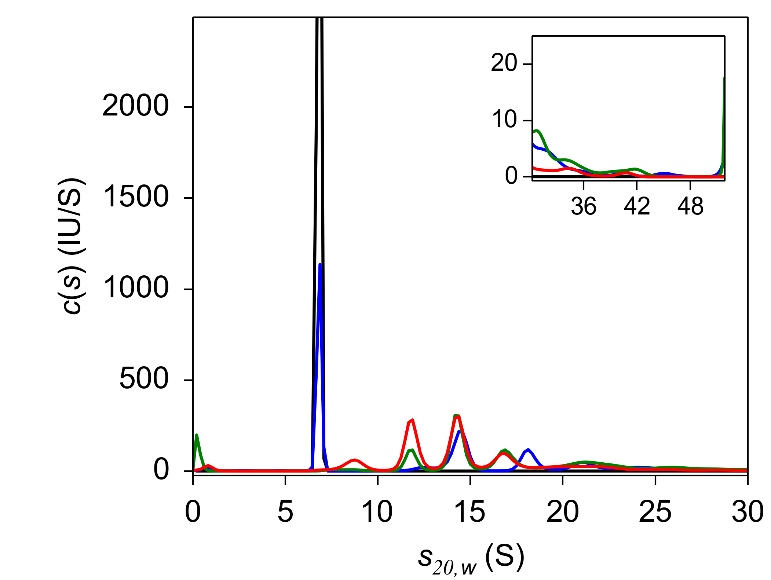

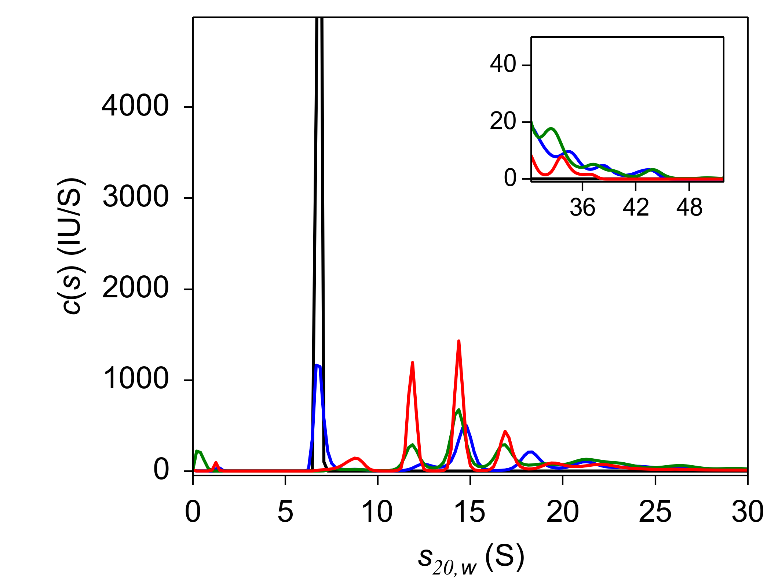

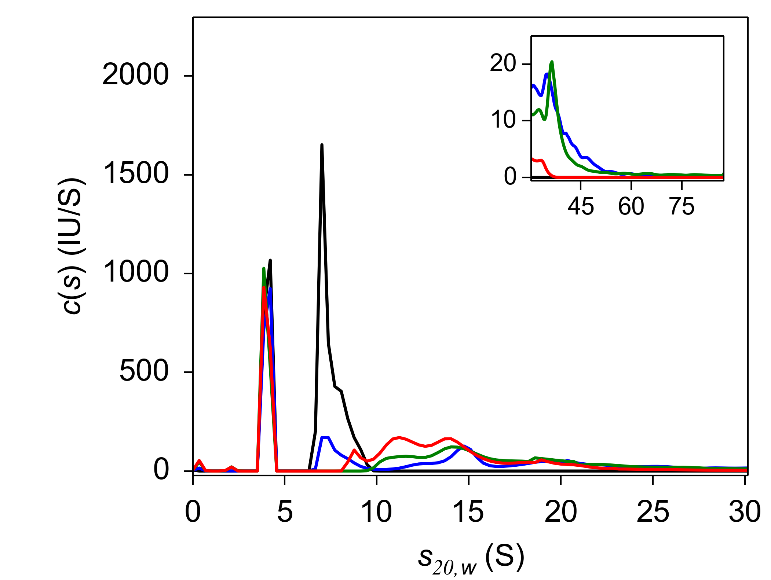

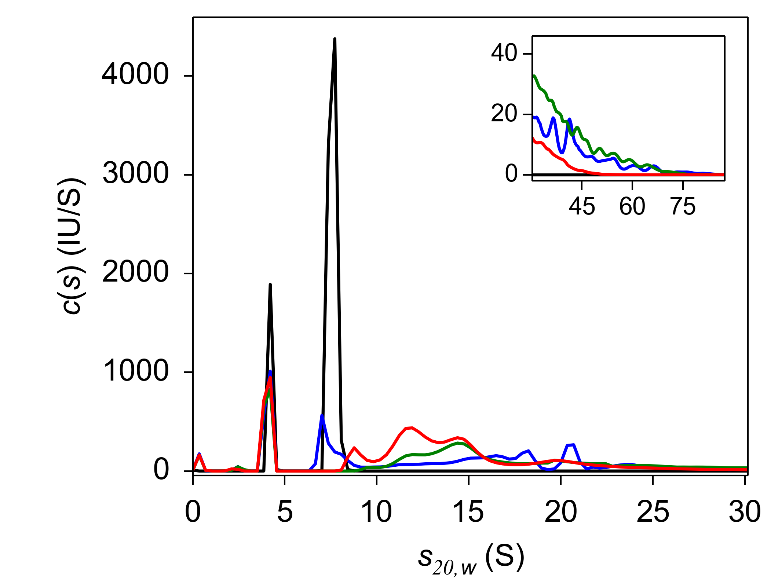


**Supplementary figure 10.** FDS-SV with 50 nM and 100 nM adalimumab in the presence of varying concentrations of TNF produced using an E. coli expression system. (**A**) 50 nM adalimumab in PBS; (**B**) 100 nM adalimumab in PBS; (**C**) 50 nM adalimumab in human serum; (**D**) 100 nM adalimumab in human serum The c(s) distributions in the absence of TNF (black) and of the 2:1 (blue), 1:1 (green), and 1:2 (red) molar mixtures of adalimumab:TNF are shown.

**A** **B**

**C** **D**

**Supplementary Table 1.** The weight-average sedimentation coefficients calculated from UV-SV experiments with 2 µM antagonists in PBS.

| TNF antagonist | TNF concentration (*µ*M) | Incubation conditions | *s_20,w_* (S) |
| --- | --- | --- | --- |
| adalimumab | 0 | 20°C, 2h | 7.1 |
|  | 2 | 20°C, 2h | 15.6 |
|  | 2 | 37°C, overnight | 14.4 |
| infliximab | 0 | 20°C, 2h | 7.9 |
|  | 2 | 20°C, 2h | 32.1 |
|  | 2 | 37°C, overnight | 27.2 |
| etanercept | 0 | 20°C, 2h | 5.2 |
|  | 2 | 20°C, 2h | 8.5 |
|  | 2 | 37°C, overnight | 8.4 |

**Supplementary Table 2.** Results of SV data analysis acquired for the 25 nM equimolar mixture of adalimumab and TNF using the “Hybrid global continuous distribution and global discrete species” model of SEDPHAT. Models were generated by sequentially assigning a reasonable MW calculated for adalimumab-TNF complexes with different stoichiometries to each discrete peak, using MWs of 148 kDa and 55 kDa for adalimumab and TNF, respectively. Model #17 provided the best fit for the experimental data based on the minimum rmsd value. Three other acceptable models, for which the rmsd values do not exceed the critical value, include models #27, 30, and 32.

| Model number | Major adalimumab-TNF complexes sedimentation coefficients and tested stoichiometries | | | | rmsd |
| --- | --- | --- | --- | --- | --- |
|  | **8.2 S** | **11.9 S** | **14.4 S** | **17.4 S** |  |
| 1 | 1:1 | 2:1 | 3:1 | 4:2 | 11.669578 |
| 2 | 1:1 | 2:2 | 3:1 | 4:2 | 11.707754 |
| 3 | 1:1 | 2:3 | 3:1 | 4:2 | 11.808722 |
| 4 | 1:1 | 2:1 | 3:2 | 4:2 | 11.725964 |
| 5 | 1:1 | 2:1 | 3:3 | 4:2 | 11.820783 |
| 6 | 1:1 | 2:1 | 3:4 | 4:2 | 11.941706 |
| 7 | 1:2 | 2:1 | 3:1 | 4:2 | 11.754489 |
| 8 | 1:2 | 2:2 | 3:1 | 4:2 | 11.799543 |
| 9 | 1:2 | 2:3 | 3:1 | 4:2 | 11.906330 |
| 10 | 1:2 | 2:1 | 3:2 | 4:2 | 11.807510 |
| 11 | 1:2 | 2:2 | 3:2 | 4:2 | 11.839393 |
| 12 | 1:2 | 2:2 | 3:2 | 4:3 | 11.892045 |
| 13 | 1:2 | 2:1 | 3:3 | 4:2 | 11.899132 |
| 14 | 1:1 | 2:1 | 3:1 | 4:3 | 11.722467 |
| 15 | 1:1 | 2:1 | 3:1 | 4:4 | 11.775784 |
| 16 | 1:1 | 2:1 | 3:1 | 4:5 | 11.828881 |
| 17 | 1:1 | 2:1 | 3:1 | 3:2 | 11.544723 |
| 18 | 1:1 | 2:1 | 3:1 | 3:3 | 11.587009 |
| 19 | 1:1 | 2:2 | 3:1 | 3:2 | 11.591480 |
| 20 | 1:1 | 2:2 | 3:1 | 3:3 | 11.630110 |
| 21 | 1:1 | 2:2 | 3:2 | 3:3 | 11.669718 |
| 22 | 1:1 | 2:3 | 3:1 | 3:2 | 11.701136 |
| 23 | 1:2 | 2:1 | 3:1 | 3:2 | 11.629520 |
| 24 | 1:2 | 2:2 | 3:1 | 3:2 | 11.683203 |
| 25 | 1:2 | 2:2 | 3:2 | 3:3 | 11.758193 |
| 26 | 1:2 | 2:3 | 3:1 | 3:2 | 11.798700 |
| 27 | 1:1 | 2:1 | 2:2 | 3:1 | 11.567940 |
| 28 | 1:1 | 2:1 | 2:2 | 3:2 | 11.600539 |
| 29 | 1:1 | 2:1 | 2:3 | 3:1 | 11.693245 |
| 30 | 1:1 | 2:2 | 2:3 | 3:1 | 11.568986 |
| 31 | 1:1 | 2:2 | 2:3 | 3:2 | 11.597495 |
| 32 | 1:1 | 2:1 | 2:2 | 2:3 | 11.552262 |
| 33 | 1:2 | 2:1 | 2:2 | 2:3 | 11.642417 |
| 34 | 1:2 | 2:1 | 2:2 | 3:1 | 11.658317 |
| 35 | 1:2 | 2:1 | 2:2 | 3:2 | 11.691151 |
| 35 | 1:2 | 2:2 | 2:3 | 3:1 | 11.662861 |
| 36 | 1:2 | 2:1 | 2:3 | 3:2 | 11.627912 |
| 37 | 1:2 | 2:2 | 2:3 | 3:1 | 11.662861 |
| 38 | 1:1 | 1:2 | 2:1 | 2:2 | 11.779770 |
| 39 | 1:1 | 1:2 | 2:1 | 2:3 | 11.798127 |
| 40 | 1:1 | 2:2 | 2:3 | 3:3 | 11.635179 |
| 41 | 1:1 | 2:2 | 2:3 | 3:2 | 11.597495 |
| 42 | 1:1 | 1:2 | 2:2 | 3:2 | 11.743378 |

**Supplementary Table 3.** Results of SV data analysis acquired for the 25 nM equimolar mixture of infliximab and TNF using the “Hybrid global continuous distribution and global discrete species” model of SEDPHAT. Models were generated by sequentially assigning a reasonable MW calculated for infliximab-TNF complexes with different stoichiometries to each discrete peak, using MWs of 149 kDa and 55 kDa for infliximab and TNF, respectively. Model #17 provided the best fit for the experimental data based on the minimum rmsd value. Five other acceptable models, for which the rmsd values do not exceed the critical value, include models #1, 18, 23, 29, and 43.

| Model number | Major infliximab-TNF complexes sedimentation coefficients and tested stoichiometries | | | | rmsd |
| --- | --- | --- | --- | --- | --- |
|  | **8.2 S** | **11.9 S** | **14.4 S** | **17.4 S** |  |
| 1 | 1:1 | 2:1 | 3:1 | 4:2 | 10.813511 |
| 2 | 1:1 | 2:2 | 3:1 | 4:2 | 10.852129 |
| 3 | 1:1 | 2:3 | 3:1 | 4:2 | 10.916999 |
| 4 | 1:1 | 2:1 | 3:2 | 4:2 | 10.828753 |
| 5 | 1:1 | 2:1 | 3:3 | 4:2 | 10.861959 |
| 6 | 1:1 | 2:1 | 3:4 | 4:2 | 10.907250 |
| 7 | 1:2 | 2:1 | 3:1 | 4:2 | 10.839237 |
| 8 | 1:2 | 2:2 | 3:1 | 4:2 | 10.881311 |
| 9 | 1:2 | 2:3 | 3:1 | 4:2 | 10.949172 |
| 10 | 1:2 | 2:1 | 3:2 | 4:2 | 10.853445 |
| 11 | 1:2 | 2:2 | 3:2 | 4:2 | 10.889827 |
| 12 | 1:2 | 2:2 | 3:2 | 4:3 | 10.904376 |
| 13 | 1:2 | 2:1 | 3:3 | 4:2 | 10.885724 |
| 14 | 1:1 | 2:1 | 3:1 | 4:3 | 10.828730 |
| 15 | 1:1 | 2:1 | 3:1 | 4:4 | 10.845752 |
| 16 | 1:1 | 2:1 | 3:1 | 4:5 | 10.863925 |
| 17 | 1:1 | 2:1 | 3:1 | 3:2 | 10.789126 |
| 18 | 1:1 | 2:1 | 3:1 | 3:3 | 10.794169 |
| 19 | 1:1 | 2:2 | 3:1 | 3:2 | 10.832059 |
| 20 | 1:1 | 2:2 | 3:1 | 3:3 | 10.835287 |
| 21 | 1:1 | 2:2 | 3:2 | 3:3 | 10.844739 |
| 22 | 1:1 | 2:3 | 3:1 | 3:2 | 10.901054 |
| 23 | 1:2 | 2:1 | 3:1 | 3:2 | 10.814472 |
| 24 | 1:2 | 2:2 | 3:1 | 3:2 | 10.860865 |
| 25 | 1:2 | 2:2 | 3:2 | 3:3 | 10.872695 |
| 26 | 1:2 | 2:3 | 3:1 | 3:2 | 10.932860 |
| 27 | 1:1 | 2:1 | 2:2 | 3:1 | 10.826286 |
| 28 | 1:1 | 2:1 | 2:2 | 3:2 | 10.826851 |
| 29 | 1:1 | 2:1 | 2:3 | 3:1 | 10.793848 |
| 30 | 1:1 | 2:2 | 2:3 | 3:1 | 10.843163 |
| 31 | 1:1 | 2:2 | 2:3 | 3:2 | 10.840069 |
| 32 | 1:1 | 2:1 | 2:2 | 2:3 | 10.830648 |
| 33 | 1:2 | 2:1 | 2:2 | 2:3 | 10.857592 |
| 34 | 1:2 | 2:1 | 2:2 | 3:1 | 10.853390 |
| 35 | 1:2 | 2:1 | 2:2 | 3:2 | 10.854157 |
| 35 | 1:2 | 2:2 | 2:3 | 3:1 | 10.872537 |
| 36 | 1:2 | 2:1 | 2:3 | 3:2 | 10.818920 |
| 37 | 1:2 | 2:2 | 2:3 | 3:1 | 10.872537 |
| 38 | 1:1 | 1:2 | 2:1 | 2:2 | 10.910799 |
| 39 | 1:1 | 1:2 | 2:1 | 2:3 | 10.902935 |
| 40 | 1:1 | 2:2 | 2:3 | 3:3 | 10.843797 |
| 41 | 1:1 | 2:2 | 2:3 | 3:2 | 10.840069 |
| 42 | 1:1 | 1:2 | 2:2 | 3:2 | 10.852136 |
| 43 | 1:1 | 2:1 | 3:2 | 3:3 | 10.809174 |
| 44 | 1:1 | 2:1 | 3:3 | 3:4 | 10.852148 |
| 45 | 1:1 | 2:1 | 3:2 | 3:4 | 10.819331 |
| 46 | 1:2 | 2:1 | 2:3 | 3:1 | 10.819765 |

**Supplementary Table 4.** The weight-average sedimentation coefficients calculated for 50 nM and 100 nM adalimumab in the presence of different amounts of TNF in PBS and human serum.

| adalimumab concentration (nM) | TNF concentration (nM) | *s_20,w_* (S) | |
| --- | --- | --- | --- |
|  |  | PBS | human serum^1^ |
| 50 | 0 | 6.8 | 7.5 |
|  | 25 | 12.9 | 16.0 |
|  | 50 | 16.7 | 21.8 |
|  | 100 | 14.2 | 15.8 |
| 100 | 0 | 6.8 | 7.6 |
|  | 50 | 13.7 | 16.0 |
|  | 100 | 17.7 | 23.2 |
|  | 200 | 14.3 | 16.3 |

^1^The signal originating from the HSA-bilirubin complex was subtracted from the total fluorescent signal.

**Supplementary Table 5.** The weight-average sedimentation coefficients calculated for 25 nM adalimumab in the presence of different amounts of TNF in PBS solutions containing approximately 40 mg/mL HSA.

| TNF concentration (nM) | *s_20,w_* (S) |
| --- | --- |
| 0.0 | 6.2 |
| 2.5 | 7.4 |
| 12.5 | 15.4 |
| 25.0 | 13.1 |
